# Rab11Bis required for binding and entry of recent H3N2, but not H1N1, influenza A isolates

**DOI:** 10.1128/jvi.02111-25

**Published:** 2026-06-02

**Authors:** Allyson H. Turner, Sara A. Jaffrani, Hannah C. Kubinski, Deborah P. Ajayi, Matthew B. Owens, Conor D. Fanuele, Madeline P. McTigue, Cailey L. Appenzeller, Addington Bowling, Hannah W. Despres, Madaline M. Schmidt, David J. Shirley, Jessica W. Crothers, Ramiro Barrantes-Reynolds, Emily A. Bruce

**Affiliations:** 1 Department of Microbiology and Molecular Genetics, University of Vermont, Burlington, Vermont, USA; 2 Cellular, Molecular, and Biomedical Sciences Graduate Program, University of Vermont Larner College of Medicine 12352 , Burlington, Vermont, USA; 3 Data Science Department, Faraday, Inc., Burlington, Vermont, USA; 4 Department of Pathology and Laboratory Medicine, University of Vermont 2092, Burlington, Vermont, USA; Fred Hutchinson Cancer Center Vaccine and Infectious Disease Division, Seattle, Washington, USA

**Keywords:** H3N2, entry, Rab11B, Rab11A, influenza, receptor

## Abstract

Influenza A virus (IAV) depends on host proteins to complete several important functions, including trafficking viral proteins throughout the cell. Previous work has established a critical role for the cellular vesicular trafficking protein, Rab11A, in transporting the viral genome segments to the site of budding at the plasma membrane. While the role of Rab11A in IAV assembly is relatively well understood, very little is known about the function of a closely related isoform (Rab11B) during influenza virus infection. We have shown that both Rab11A and Rab11B are required for successful IAV infection by current H1N1 or H3N2 isolates. Cells in which either Rab11A or Rab11B were depleted failed to efficiently produce virus, with significant reductions in infectious titer. Surprisingly, our data revealed that recent (2022) H3N2, but not H1N1, isolates failed to efficiently produce viral proteins in single-cycle infections when Rab11B (but not Rab11A) was depleted. Flow cytometry analysis suggests that the defect in protein production is driven by a reduction in the total number of infected cells, rather than a decrease in viral protein production at the single-cell level. Using reverse genetics and “7+1” reassortant viruses, we mapped this Rab11B-dependent early defect in recent H3N2 isolates to the HA gene. RT-qPCR analysis of H3N2 virions bound to the cell surface showed a ~50% decrease in virus binding to the surface of cells depleted of Rab11B, but not Rab11A. Analysis of cell surface α2,6 and α2,3 sialic acids revealed no significant global change in sialic acid profile upon the depletion of Rab11B. As H3N2 virions could be removed by exogenous neuraminidase, the totality of these data suggests that the H3N2 failure to bind is the result of a loss of one (or more) specific sialylated cell surface protein(s) upon Rab11B depletion, rather than a decrease in bulk α2,6 sialic acid levels. These data suggest a novel role for Rab11B during viral entry that is specific to H3N2 isolates.

**IMPORTANCE:**

Influenza A is a major human pathogen, which poses risks through both the continuous circulation of “seasonal” influenza viruses (H1N1 and H3N2 subtypes) as well as the emergence of novel pandemic strains from animal hosts. Here, we demonstrate that contemporaneous H3N2 (but not H1N1) subtypes enter human lung cells through a Rab11B-dependent mechanism. This is distinct from the well-known role of Rab11A later in the life cycle, where it mediates the transport of viral ribonucleoprotein complexes to the site of virion assembly. These findings are relevant for assessing the risk that recently emerged zoonotic influenza viruses can enter human lung cells. Our work suggests that H1N1 and H3N2 viruses enter via different routes, which are not dependent on sialic acid levels. Our data provide important foundational information for the growing number of Rab11-dependent viruses, as it suggests that the Rab11 isoforms can affect both viral entry and viral exit.

## INTRODUCTION

Influenza A virus (IAV) is a highly infectious pathogen, causing seasonal epidemics that result in around 3–5 million severe cases each year, in addition to causing periodic pandemics that emerge from novel IAV strains (1, 2). While there are many different subtypes of IAV, currently, there are only two which are circulating within the human population, H1N1 and H3N2 (3). Influenza owes much of its pandemic potential to the fact that its genome is composed of eight distinct segments, which can mix to form novel genetic combinations in a cell infected with two influenza viruses simultaneously, a process known as reassortment (4).

As a segmented negative-strand RNA virus, influenza encodes up to 14 known proteins on its eight segments (3). After transcription and translation, these proteins collectively carry out the functions required to complete the viral life cycle within a host cell. Cellular entry of the influenza virion is mediated by the viral glycoprotein hemagglutinin (HA) binding to sialic acid moieties on the cell surface, which is thought to trigger receptor-mediated endocytosis (5, 6). Broadly speaking, influenza viruses that infect human cells are thought to bind to α2,6 sialic acid modifications on cell surface proteins, differentiating them from avian viruses, which are thought to prefer α2,3 sialic acids (3, 4, 7). The classical model of influenza binding continues to be refined and expanded; however, through new work which suggests that there may be subtype-specific effects, and both the identity of the sialylated protein and its cell surface density may play important roles (8–15). Specific characteristics of the sialic acid modifications themselves can also affect binding, with more recent H3N2 isolates preferring a highly branched structure (16). Intriguingly, H3N2s have shown decreased avidity for α2,6 sialic acid from 1968 to 2010, which correlates with a decrease in infection severity and evidence that NA contributed to mediating receptor binding (17). After viral binding, internalization occurs through an incompletely understood process that is likely to involve signaling from one or more cell surface proteins and may require specific “internalization receptors”(18, 19).

After endocytosis, the lowered pH of the endosome triggers a conformational change in HA, which mediates fusion between viral and cellular membranes (20–23). The acidic environment of the endosome also triggers activation of the matrix 2 protein (M2) ion channel, which transports protons to the interior of the virion, resulting in dissociation of M1 from the ribonucleoprotein complexes (RNPs) that is required for their subsequent nuclear import (24). Once released into the cytoplasm, the RNP complexes that comprise the genome segments are transported to the nucleus, where influenza genome replication occurs. During late stages of viral infection, newly produced genome segments (vRNPS) comprising full-length negative-sense RNA molecules are coated with nucleoprotein (NP) and bound to the polymerase complex composed of the polymerase basic protein 1 (PB1), polymerase basic protein 2 (PB2), and the polymerase acidic protein (PA) and then exported from the nucleus. Once in the cytoplasm, vRNPs must traffic to the site of budding at the plasma membrane before being released in newly formed virions to start the cycle of infection anew (25).

The process of transporting the vRNPs during this stage is highly dependent on the cellular Rab11 pathway, which is co-opted during viral infection to coordinate the apical transport of the influenza genome (26–28). Rab11 is a member of the Rab GTPase family and plays a key role in vesicular transport within the recycling endosome pathway (29). A direct interaction between Rab11A and PB2 (a component of the influenza tripartite polymerase complex) mediates binding between Rab11A and negative-sense vRNPs, with no binding observed to full-length positive-sense or viral mRNA species (26, 30). This interaction is hypothesized to disrupt the binding of Rab11-family interacting proteins (FIPs), possibly through Rab11’s regulatory Switch I region (30). Influenza infection disrupts the normal functioning of the endosomal trafficking pathway to favor transport of vRNPs to the plasma membrane (31–33). During transport, large clusters of multiple genome segments that colocalize with Rab11 can be observed by fluorescent *in situ* hybridization (26). This Rab11-dependent transport serves to concentrate and cluster the eight IAV segments in defined subcellular sites, dependent on microtubules (34–39). Finally, Rab11 is known to play a role in the very late stages of the viral life cycle. Rab11 can traffic vRNPs between cells through tunneling nanotubules, allowing for cell-to-cell spread without the generation of complete virions (40). Cells lacking Rab11 fail to produce filamentous virions and exhibit signs of budding defects, possibly due to a secondary failure to transport the viral genome to the site of budding (41).

Notably, the majority of this research has been focused exclusively on the role of Rab11A, while a small subset of studies looked at the combined effect of Rab11A and Rab11B. Studies examining the role of the different Rab11 isotypes, in the context of cancer, reveal that the two isoforms can play distinct roles, leading us to wonder if there might also be differences in how they interact with viral proteins during the IAV lifecycle (42). While the exact binding site between PB2 and Rab11A is unknown, the interaction between these proteins is completely abolished by mutations to the C-terminal Class I switch region, an area that is highly conserved between the different Rab11 family members (30). These data, along with a previously reported drop in titer from A549 cells depleted of Rab11B and infected with PR8, suggested to us that Rab11B might play a similar role as Rab11A during influenza infection, although this phenotype did not extend to HEK-293T cells (41). As the majority of studies examining the role of Rab11 in influenza infection have utilized older (often lab-adapted) strains, we were also curious about whether the requirement for Rab11 extended to currently circulating strains of influenza A. Here, we show that both Rab11A and Rab11B are required for the efficient production of infectious virions from seasonal H1N1 and H3N2 subtypes circulating in 2022. As expected, Rab11A played a role late in infection (i.e., post-protein production) for both subtypes. Surprisingly, we discovered that Rab11B is required early in the life cycle of H3N2 infection, with cells lacking Rab11B failing to efficiently produce viral proteins. We were able to map this defect specifically to the HA gene and trace it to a defect early in viral entry, as H3N2 (but not H1N1) virions fail to bind to the surface of cells lacking Rab11B. Our findings suggest a surprising new role for one of the two highly conserved Rab11 isoforms in influenza infection and highlight a clear role of differential requirements for viral binding and entry of recent H1N1 vs. H3N2 subtypes.

## RESULTS

**Fig 1:**
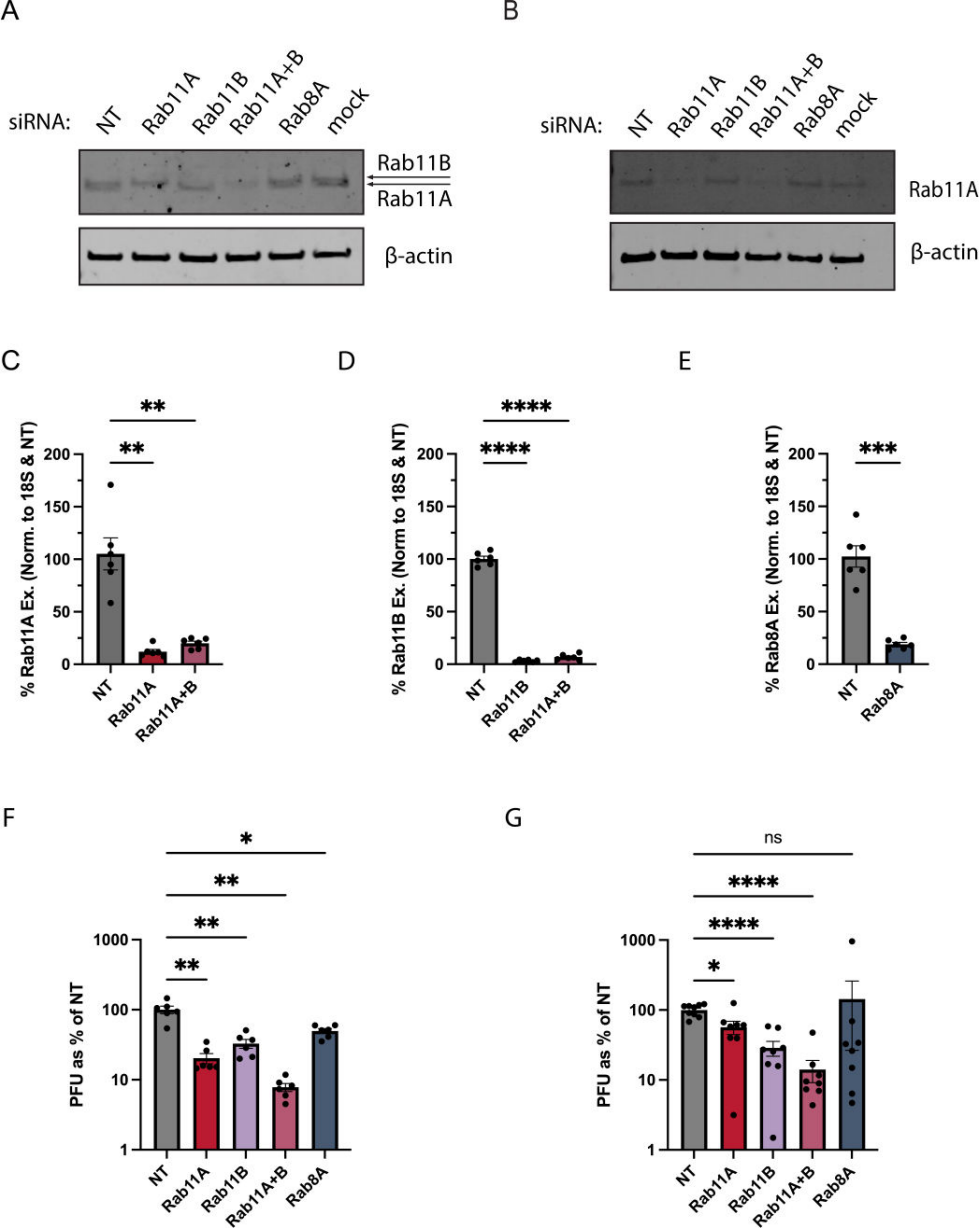
Rab11A and Rab11B are required for the replication of recent H1N1 and H3N2 influenza A isolates. A549 cells were treated with siRNAs targeting Rab11A, Rab11B (singly and in combination), Rab8A, or a non-targeting control; 48 h post-transfection,the cells were harvested to determine protein (**A, B**)or RNA (**C–E**)expression. (**A**)Total Rab11 (Rab11A plus Rab11B) or (**B**)Rab11A expression was detected by western blot using either non-specific or Rab11A-specific isoform antibodies. Relative Rab11A (**C**),Rab11B (**D**),or Rab8A (**E**)expression was determined by RT-qPCR, using 18S as a housekeeping gene and normalized to the NT average of each biological experiment. Alternatively, at 48 hpt,the cells were infected with (**F**)a 2022 IAV isolate of H1N1 (A/Burlington/UVM-0478/2022) or (G) H3N2 (A/Burlington/UVM-1927/2022) subtype at an MOI of 1. Viral supernatants were collected at 16 hpi and titered by plaque assay (PFU; plaque-forming unit). Mean +/−SEMof relative PFU (normalized to the NT average of each biological experiment) is plotted. Statistical comparisons (**C, D, F, G**)were done using Welch’s one-way ANOVA with Dunnett’s multiple comparisons(*[*P*<0.05], **[*P*<0.01], ***[*P*<0.001], ****[*P*<0.0001]) and Welch’s *t*-test (**E**).*N*=6from three biological experiments (**C–F**)and *N*=8from four biological experiments (**G**)are shown.

To determine the effect Rab11A and Rab11B played on the replication of current seasonal influenza viruses, we used siRNAs targeting each gene to transiently knockdown expression in human adenocarcinoma alveolar basal epithelial (A549) cells, in the hopes of minimizing off-target effects that can occur with stable knockdown/knockout. In addition to Rab11A and Rab11B, we included a third Rab (Rab8A) that is also involved in recycling endosome trafficking but is not known to impact influenza viral replication, as well as a non-targeting control. To verify the efficiency of our knockdown, we initially used western blot to measure Rab11 protein expression, relative to a housekeeping gene (β-actin). We observed global Rab11 protein depletion using a non-isoform-specific antibody in cells treated with siRNA targeting both Rab11A and Rab11B (Fig. 1A). Using a Rab11A isoform-specific antibody, we could see the depletion of Rab11A in cells treated with siRNAs to Rab11A (or Rab11A and B in combination) (Fig. 1B). However, due to difficulties in antibody sensitivity when detecting endogenous protein levels, we used RT-qPCR to quantify gene expression, relative to a house keeping transcript (18S) and expression in a NT control at 48 h post-transfection. Our RT-qPCR results verified Rab11A gene expression was reduced to about 15% of endogenous levels (Fig. 1C), Rab11B to ~5% (Fig. 1D), and Rab8 to ~20% (Fig. 1E). For cells in which we simultaneously depleted Rab11A and Rab11B, we used the same total amount of RNA (i.e., half the level/gene for a single knockdown) to avoid cell toxicity. In this case, depletions were slightly less efficient, with Rab11A gene expression reduced to ~20% (Fig. 1C) and Rab11B to ~10% (Fig. 1D).

Having confirmed the efficiency of our depletions, we then challenged each depleted condition using two different, recent, low-passage IAV isolates, A/Burlington/UVM-0478/2022 (H1N1) (denoted in this study as UVM-0478) and A/Burlington/UVM-1927/2022 (H3N2) (denoted here as UVM-1927) to perform single-cycle infections. As predicted, we found that cells in which either or both Rab11A/Rab11B were depleted resulted in a significant reduction in PFU of virus produced during infection. We saw a ~3-fold to 5-fold reduction in viral titer in cells lacking Rab11A or Rab11B and a further drop in cells lacking Rab11A+B(7-fold to 10-fold), in cells infected with either UVM-0478 (Fig. 1F) or UVM-1927 (Fig. 1G). In cells depleted of Rab8A and infected with UVM-0478, we saw a small but consistent drop in titer (~2-fold), while UVM-1927 replication was unaffected (raw viral titers in Fig. S1).

**Fig 2:**
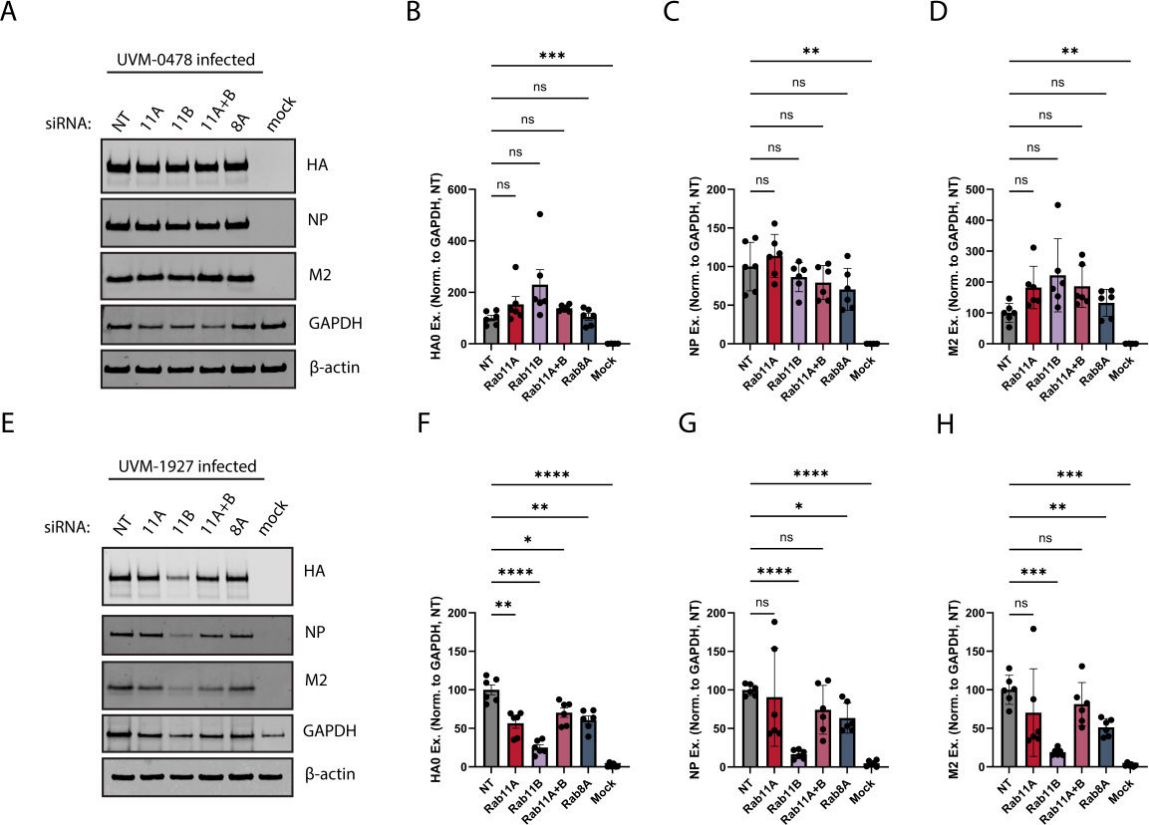
Rab11B is required early in the life cycle of a recent H3N2 IAV isolate. A549 cells were treated with siRNAs targeting Rab11A, Rab11B (singly and in combination), Rab8A, or a non-targeting control; 48 hpt, cells were infected with (**A**)2022 IAV isolate of H1N1 (A/Burlington/UVM-0478/2022) at an MOI of 1 or mock-infected. At 16 hpi, cell lysates were collected and visualized by SDS-PAGE and western blot using rabbit anti-HA and anti-GAPDH antibodies in addition to mouse anti-NP and anti-M2 antibodies. Expression of (**B**)HA, (**C**)NP, and (**D**)M2 was quantified and normalized to GAPDH levels and the NT control. Alternatively, cells were infected with (**E**)2022 IAV isolate of H3N2 (A/Burlington/UVM-1927/2022) at an MOI of 1 or mock-infected; 16 hpi, cell lysates were collected and visualized by SDS-PAGE and western blot using rabbit anti-HA and anti-GAPDH antibodies in addition to mouse anti-NP and anti-M2 antibodies. Expression of (**F**)HA, (**G**)NP, and (**H**)M2 was quantified and normalized to GAPDH levels and the average NT control for each biological replicate; quantification from six technical replicates (three biological experiments) is shown. Mean +/−SEMis plotted. Statistical comparisons (**B–D, F–H**)were done using the Welch’s one-way ANOVA with Dunnett’s multiple comparisons (*[*P*<0.05], **[*P*<0.01], ***[*P*<0.001], ****[*P*<0.0001]).

To verify the drop in titer observed was due to the previously characterized role of Rab11 in late-stage RNP trafficking, we then examined the viral protein production of infected cells in our knockdown conditions. We observed no significant effect on viral protein expression in cells infected with UVM-0478 (Fig. 2A) after densitometry quantification was used to determine the levels of HA0, NP, or M2 (Fig. 2B through D) normalized to the cellular housekeeping gene, GAPDH (or alternatively, normalized to β-actin, Fig. S2). Unexpectedly, we observed a significant drop in viral protein production in cells depleted of Rab11B that were infected with UVM-1927 (Fig. 2E). Expression of HA0, NP, and M2 (Fig. 2F through H) was reduced to 25% of that seen in cells treated with the NT control. Surprisingly, this defect in viral protein production was partially rescued in cells simultaneously depleted of Rab11A and Rab11B. In order to determine if this rescue was due to less efficient knockdown (as cells treated with siRNA to Rab11A and B simultaneously received half the total siRNA that was used in individual knockdowns), we treated cells with a half dose of Rab11A or Rab11B siRNA (along with a non-targeting control) before infecting them with UVM-1927 as above. The failure to produce viral proteins was not affected by lowering the siRNA dose, with cells lacking Rab11B once again producing 80%–90% less HA, NP, and M2 (Fig. S3). Given prior reports of reciprocal functions in the Rab11A and Rab11B isoforms, this finding suggests that Rab11A and B could also be playing opposing functions in the context of H3N2 entry (42, 43).

**Fig 3:**
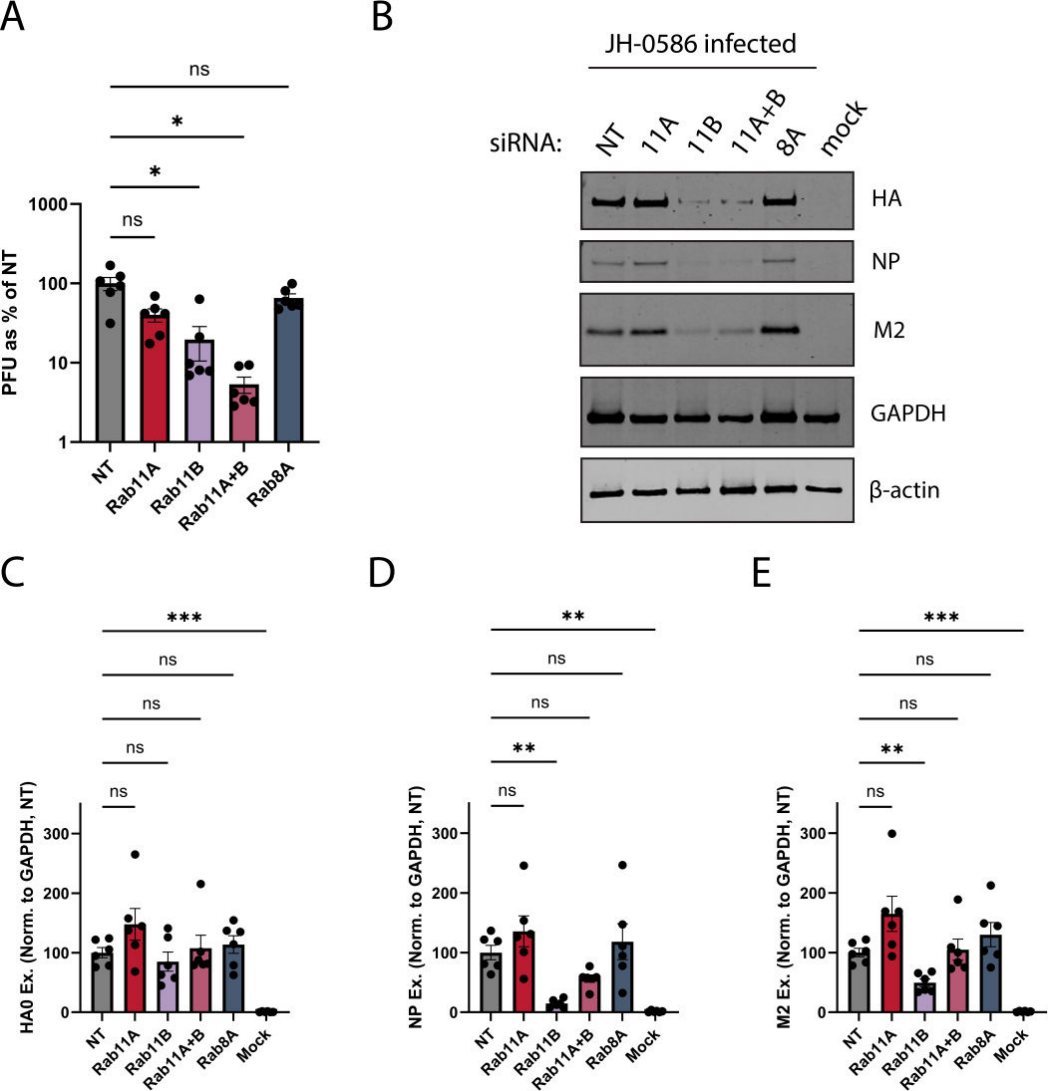
Multiple H3N2 isolates depend on Rab11B early in IAV infection. A549 cells were treated with siRNAs targeting Rab11A, Rab11B (singly and in combination), Rab8A, or a non-targeting control; 48 hpt, the cells were infected with 2022 IAV isolate of H3N2 (A/Baltimore/JH-0586/2022) at an MOI of 1 or mock-infected. (**A**)At 16 hpi, viral supernatants were collected and titered by plaque assay (PFU). (**B**)Simultaneously, cell lysates were collected and visualized by SDS-PAGE and western blot using rabbit anti-HA and anti-GAPDH antibodies in addition to mouse anti-NP and anti-M2 antibodies. Expression of viral proteins was quantified and normalized to GAPDH levels, and the average of the NT controls for each biological replicate is shown for (**C**)HA0, (**D**)NP, and (**E**)M2. Mean +/−SEMis plotted, normalized to the average of the NT control in each biological experiment.*N*=6from three biological experiments. Statistical comparisons (**A, C, D, E**)were done using Welch’s one-way ANOVA with Dunnett’s multiple comparisons (*[*P*<0.05], **[*P*<0.01], ***[*P*<0.001]).

To broaden our conclusions, we obtained a second 2022 H3N2 from a different geographical region, A/Baltimore/JH-0586/2022 (H3N2) (referred to in this study as JH-0586). JH-0586 is genetically distinct from UVM-1927, with more than 100 nucleotide-level differences across the eight segments of the two viruses. Like UVM-1927, JH-0586 also depended on the Rab11 isoforms to produce infectious virus, with a particular dependence on Rab11B, as observed above with UVM-1927 (Fig. 3A). The loss of Rab11B resulted in a 5-fold drop in viral titer, while the simultaneous depletion of Rab11A and Rab11B resulted in a 20-fold decrease,and Rab8A depletion did not affect viral replication. In line with our prior observations, viral protein production was also substantially decreased in cells lacking Rab11B (Fig. 3B). Quantification revealed that this trend was most pronounced in expression of NP (85% of NT) and M2 (~50%), while HA0 expression was decreased to a lesser extent (~15%) (Fig. 3C through E). Simultaneous depletion of Rab11A and Rab11B again appeared to, at least partially, rescue the defect induced by the loss of Rab11B alone. Finally, Rab8A depletion did not alter the expression of viral protein levels, compared to the NT control.

**Fig 4:**
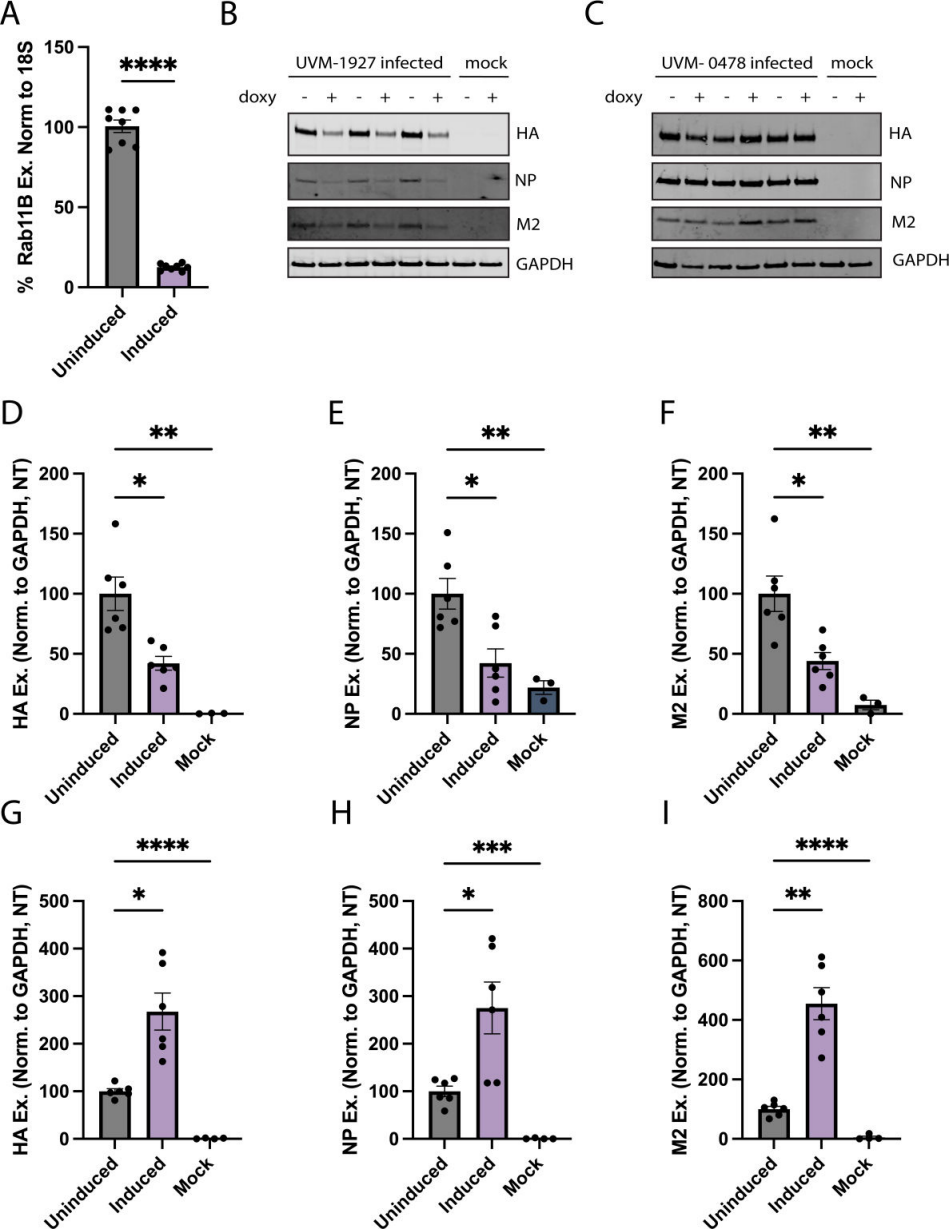
Rab11B is required early in the H3N2 life cycle in club (H441) cells. (**A**)H441 cells were transduced with a lentivirus to stably express an inducible shRNA targeting Rab11B. Cells were treated (induced) or not (uninduced) with doxycycline to induce shRNA expression, and 72 h later, RNA was harvested. Rab11B gene expression was determined by RT-qPCR, using 18S as a housekeeping gene and normalized to the uninduced average of each biological experiment. Alternatively, at 72 h,the cells were infected with (**B**)A/Burlington/UVM-1927/2022 (H3N2), (**C**)A/Burlington/UVM-0478/2022 (H1N1), or mock-infected; 16 hpi,the cell lysates were collected and visualized by SDS-PAGE and western blot using rabbit anti-HA and anti-GAPDH antibodies in addition to mouse anti-NP and anti-M2 antibodies. Expression of viral proteins was quantified and normalized to GAPDH levels, and the average of the uninduced controls for each biological replicate is shown for UVM-1927 (**D–F**)and UVM-0478 (**G–I**).Quantification from six technical replicates (two biological experiments) is shown. Mean +/−SEMis plotted. Statistical comparisons were done using Welch’s *t*-test (**A**)and Welch’s one-way ANOVA with Dunnett’s multiple comparisons (**D–I**)(*[*P*<0.05], **[*P*<0.01], ****[*P*<0.0001]).

To determine if this phenotype was restricted to A549 cells, we next expanded our study to include NCI-H441 (H441) cells, an immortalized human “club cell”-like line. To rule out off-target effects of the siRNA constructs/depletion strategy we had used in A549 cells, we used lentiviral transduction to create a stable cell line with a doxycycline-driven inducible shRNA targeting Rab11B. We achieved high (~90%) levels of Rab11B depletion in these cells 3 days after induction (Fig. 4A), allowing us to examine viral protein production of UVM-1927 after a single round of replication (Fig. 4B). Expression of HA, NP, and M2 was once again significantly (<50%) decreased in cells induced with Rab11B shRNA constructs (Fig. 4D through F). To validate that this phenotype was specific to our H3N2 isolate, we next infected these cells with our 2022 H1N1, UVM-0478 (Fig. 4C). We saw that the expression of HA, NP, and M2 (Fig. 4G through I) was not reduced but rather increased during infection in cells induced to deplete Rab11B, consistent with the previously characterized defect in viral exit reported for H1N1 strains lacking Rab11B (41). Overall, this suggests that the dependence of recent H3N2 isolates on Rab11B was not an artifact of either cell type or our depletion strategy.

**Fig 5:**
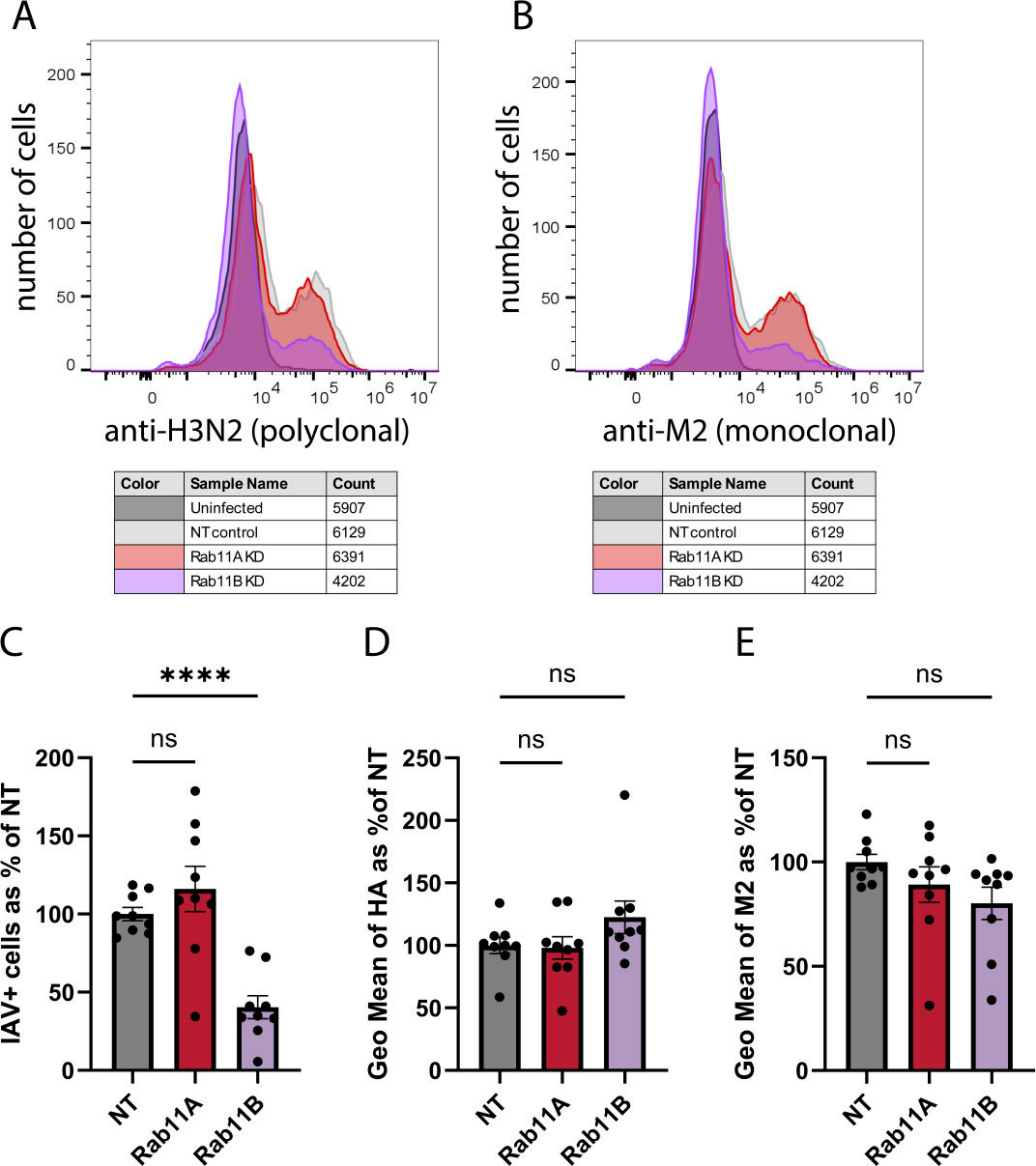
Loss of Rab11B decreases the number of H3N2 IAV-infected cells rather than their protein expression. A549 cells were treated with siRNAs targeting Rab11A, Rab11B, or a non-targeting control; 48 hpt, the cells were infected with 2022 IAV isolate of H3N2 (A/Burlington/UVM-1927/2022) at an MOI of 1 or mock-infected. At 16 hpi,the cells were washed with PBS, trypsinized, fixed, and stained for flow cytometry analysis to determine total (intracellular/extracellular) staining using (**A**)anti-H3N2 or (**B**)anti-M2 antibodies. (**C**)The percent of double HA^+^/M2^+^ cells in each experiment was determined using the mock control to determine the background staining levels. The geometric mean of (**D**)HA or (**E**)M2 expression in the infected (HA^+^/M2^+^) cell population, normalized to that of the NT-infected population, is shown. Mean +/−SEMis plotted, normalized to the average of the NT control in each biological experiment, *N*=9from three biological experiments. Statistical comparisons (**C–E**)were done using Welch’s one-way ANOVA with Dunnett’s multiple comparisons (****[*P*<0.0001]).

Next, we wanted to understand whether our phenotype was being driven by a global drop in the number of cells being infected, or if the same number of cells was infected but each produced fewer viral proteins. As western blots of total cell lysates are unsuited to analyzing protein production at a single-cell level, we used flow cytometry to measure HA and M2 expression in individual cells that had been treated with siRNAs targeting Rab11A, Rab11B, or a NT control and infected with UVM-1927 as above. The infected control cells were clearly visualized as a second peak (Fig. 5, light gray) with greater levels of staining than the mock-infected cells (dark gray, single left-most peak) after staining with both anti-H3N2 serum and an M2 antibody (Fig. 5A and B). As expected for a multiplicity of infection (MOI) of 1 (chosen due to relatively low titers of our low-passage stocks), infected cells were roughly a third of the cell population. Unsurprisingly, given our prior data, the loss of Rab11A did not alter viral protein expression patterns (Fig. 5A and B; compare maroon to light gray line). In cells lacking Rab11B, the total number of cells expressing viral proteins was reduced by ~50%, while we observed similar intensity of viral protein expression in the positive cell population (Fig. 5A and B; compare how the purple line is decreased along the *y*-axis dimension [count] but not the *x*-axis [viral antigen intensity]). Quantification of multiple biological replicates revealed a ~50%drop in the number of cells infected (i.e., producing viral proteins) when Rab11B was knocked down, compared to both the NT control and the Rab11A knockdown (Fig. 5C). Further analysis of viral protein expression within the infected cell population showed no decrease in HA or M2 expression (Fig. 5D and E). This assay revealed a significant defect in the number of Rab11B-depleted cells able to produce viral proteins, rather than a broad dampening of protein expression. This suggested that if a cell was able to make viral proteins at all, then it would produce the normal amount, implying a defect upstream of translation.

**Fig 6:**
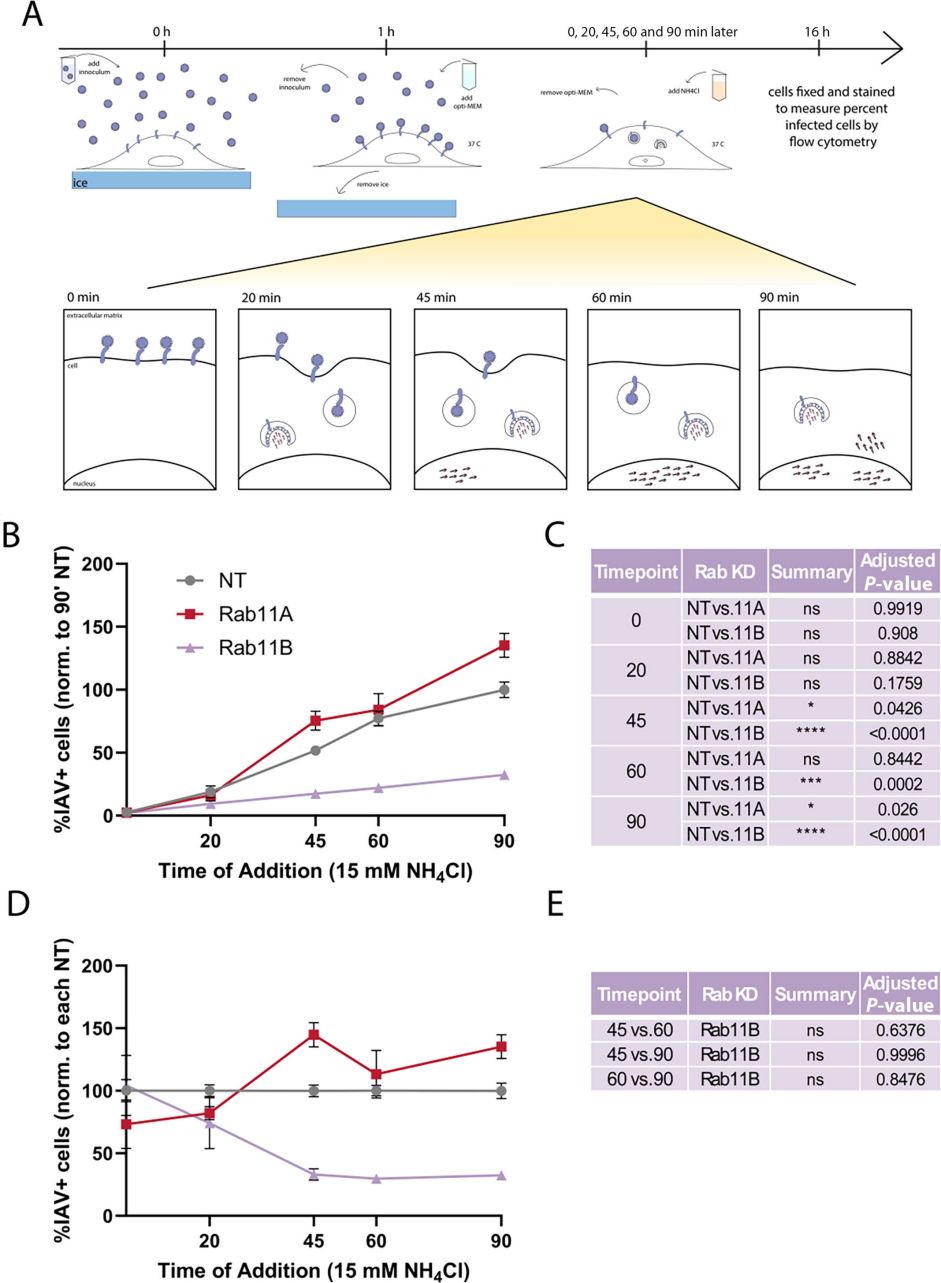
Loss of Rab11B delays the entry kinetics of the H3N2 IAV subtype. (**A**)A549 cells were treated with siRNAs targeting Rab11A, Rab11B, or a non-targeting control; 48 hpt,the cells were infected with 2022 IAV isolate of H3N2 (A/Burlington/UVM-1927/2022) at an MOI of 1 or mock-infected. At the indicated times (0, 20, 45, 60, or 90 min) post-infection, viral overlay was removed and replaced with 15 mM NH_4_Cl (to prevent endosomal acidification and thus fusion of internalized virions) for the remainder of the infection;at 16 hpi,the cells were washed with PBS, trypsinized, fixed, and stained for flow cytometry analysis to determine total (intracellular as well as extracellular) staining using anti-HA or anti-M2 antibodies. The percent of HA^+^/M2^+^ cells in each experiment was determined using the mock control to determine background staining levels. Mean +/−SEMis plotted, normalized to the average of the NT control for each biological experiment (**B**)at 90 min or (**D**)at each timepoint. (**C**)Statistical comparisons of Rab11A- or Rab11B-depleted conditions vs. the NT control were conducted using a two-way ANOVA with Dunnett’s multiple comparisons tests, and *P*-values are reported. (**E**)Statistical comparisons of the Rab11B-depleted condition over time were conducted using a two-way ANOVA with Tukey’s multiple comparisons test, and *P*-values are reported. *N*=6 replicates from three biological experiments(*[*P*<0.05], ***[*P*<0.001], ****[*P*<0.0001]).

As our data suggested that Rab11B was not acting directly on protein production, we hypothesized that the infection defect in these cells was occurring somewhere in the entry process. To look at the kinetics of entry, we used a “time of addition” assay, in which we added ammonium chloride at specific timepoints post-infection to stop endosomal acidification and thus viral fusion (44). Infections were allowed to progress in the presence of ammonium chloride, and the percentage of infected cells was measured by flow cytometry 16 h later (Fig. 6A). In cells treated with the NT control, we saw no escape from the endosome when ammonium chloride was added concurrently with viral inoculum (Fig. 6B). The majority of viral fusion appeared to occur between 20 and 60 min, consistent with previously reported half-times for influenza viral entry (44). Cells lacking Rab11A showed no defect in entry kinetics, with an equal or greater number of cells infected throughout the measured time course (Fig. 6B and C). In Rab11B-depleted cells, we observed a significant drop in the number of infected cells at 45, 60, and 90 min compared to the NT control (Fig. 6B and C). To better visualize whether the Rab11B entry kinetics were delayed compared to NT (which could imply the utilization of an alternative trafficking pathway), we normalized the percent of infected cells to those infected in the NT control at each timepoint (Fig. 6D). This revealed that cells lacking Rab11B had significant defects in their ability to support normal viral entry, but the rate of entry itself was not delayed compared to the NT control (Fig. 6D), with a near-constant drop in number of infected cells at each of the later timepoints compared to NT (Fig. 6E).

**Fig 7:**
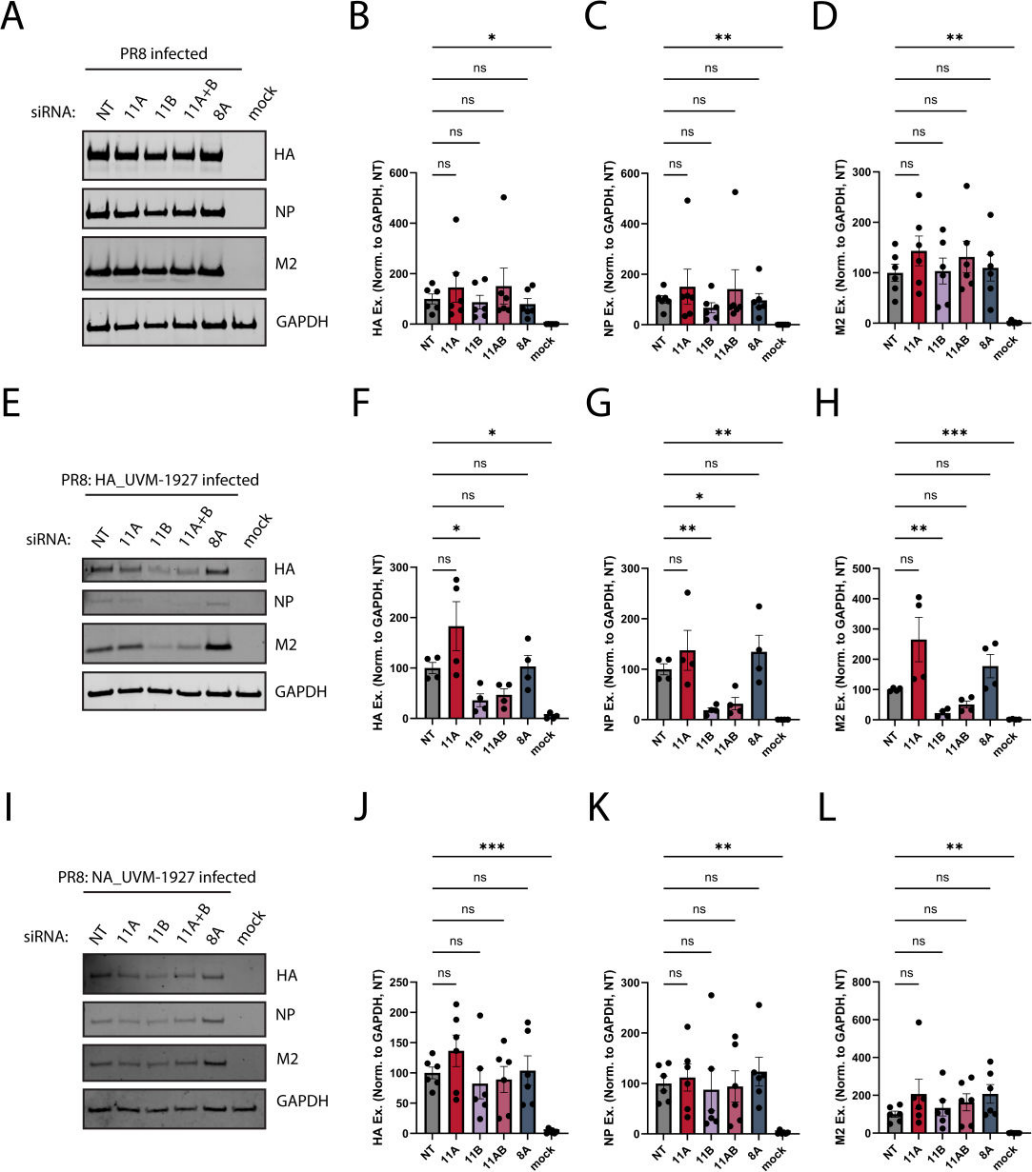
H3N2 dependence on Rab11B early in infection can be mapped to the HA gene. A549 cells were treated with siRNAs targeting Rab11A, Rab11B (singly and in combination), Rab8A, or a non-targeting control; 48 hpt, cells were infected with (**A**) A/Puerto Rico/8/1934 (PR8), (**E**)a reverse genetics-derived 7+1 reassortment containing the HA gene of UVM-1927 (PR8:HA UVM-1927), or (**I**) a reverse genetics-derived 7+1 reassortment containing the NA gene of UVM-1927 (PR8:NA UVM-1927) at an MOI of 1 or mock-infected. At 16 hpi, cell lysates were collected and visualized by SDS-PAGE and western blot using rabbit anti-HA and anti-GAPDH antibodies in addition to mouse anti-NP and anti-M2 antibodies. Expression of viral proteins was quantified and normalized to GAPDH levels, and the average of the NT controls for each biological replicate is shown for PR8 (**B–D**),PR8:HA UVM-1927 (**F–H**),and PR8:NA UVM-1927 (**J–L**). Mean +/−SEMis plotted(*[*P*<0.05], **[*P*<0.01], ***[*P*<0.001], ****[*P*<0.0001]).*N*=6from three biological experiments (**B, C, D, J, K, L**)or 4 from two biological experiments (**F–H**).Statistical comparisons were done using Welch’s one-way ANOVA with Dunnett’s multiple comparisons.

Given the profound defects we observed in overall entry, we next sought to determine which viral gene (or genes) contributed to the singular dependence of our H3N2 viruses on Rab11B. Viral binding is governed primarily by HA (with a possible role for NA), with fusion dependent on HA, uncoating relying on M2, and transport to the nucleus likely mediated by multiple genes. After first verifying that A/Puerto Rico/8/1934 (H1N1, referred to as PR8 in future) was not dependent on Rab11B for viral protein production (Fig. 7A through D), we created 7+1 reassortants of the UVM-1927 HA or NA gene segments in a PR8 background using an established reverse genetics system. The PR8 7+1 reassortant containing the UVM-1927 HA gene (PR8: HA_UVM-1927) showed a significant (75%–90%) reduction in viral protein levels when HA, NP, and M2 expression was quantified in cells lacking Rab11B (Fig. 7E through H). Conversely, no significant reduction in viral protein expression was seen in the 7+1 reassortant containing the UVM-1927 NA gene (Fig. 7I through L). These data show that the dependence on Rab11B for viral protein production can be mapped to the HA segment and to the HA protein itself, given that segment 4 is monocistronic (45). This result strongly suggested that Rab11B is required at one of the earliest stages of entry, given that HA plays key roles in binding and fusion.

**Fig 8:**
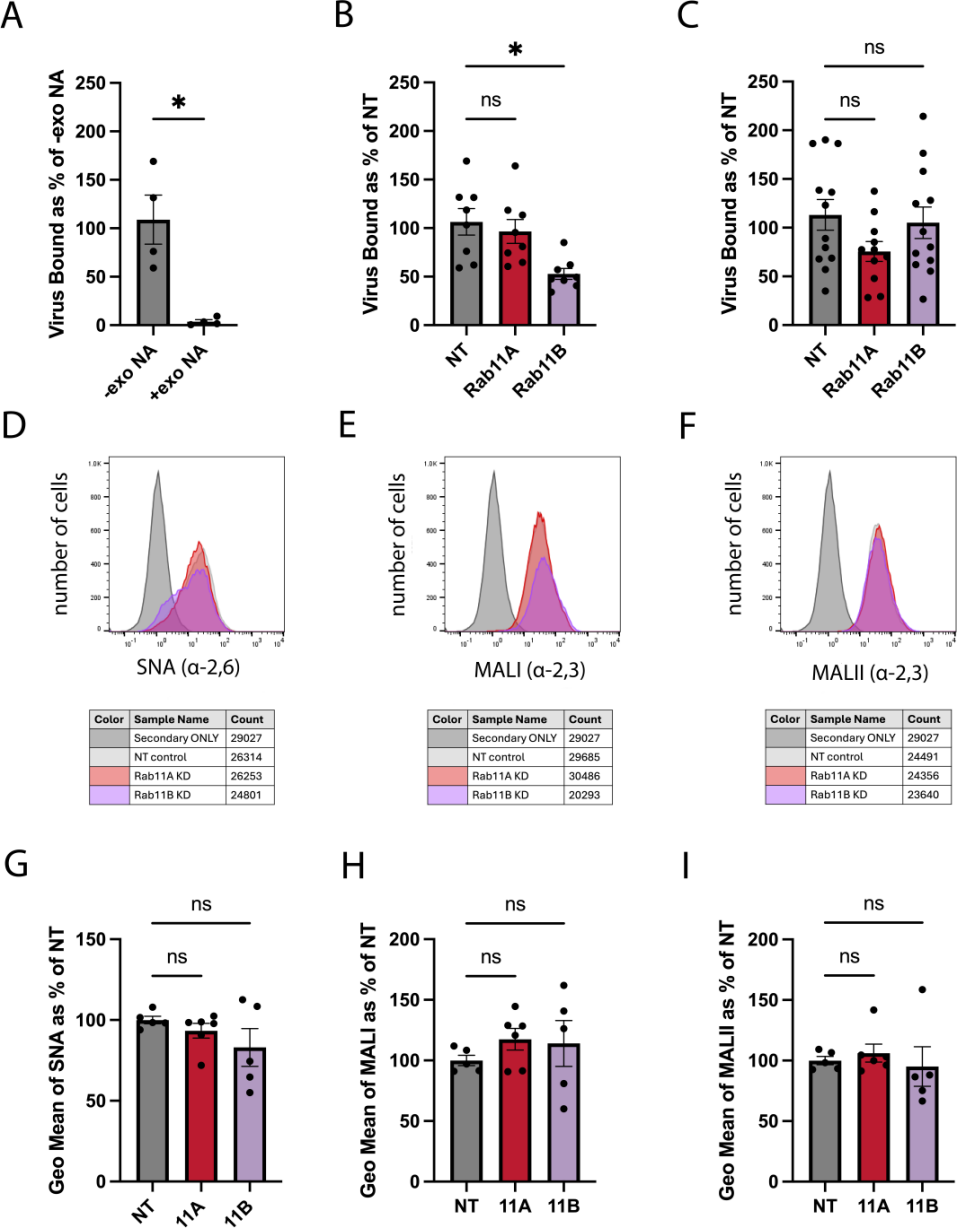
Rab11B is required for efficient binding of H3N2 but not H1N1 virions to the cell surface but does not alter global sialic acid levels. A549 cells were prechilled on ice, after which the cold virus [A/Burlington/UVM-1927/2022 (H3N2) at an MOI of 1] was bound to cells for 1 h to synchronize binding. Cells were washed three times with ice-cold PBS to remove unbound virus, and total virion binding was determined by extracting RNA from each well and using RT-qPCR to determine the relative levels of IAV RNA vs. a housekeeping control (18S). (**A**)Bound virions were stripped by treating cells with exogenous neuraminidase. Alternatively, cells were treated with siRNAs targeting Rab11A, Rab11B, or a non-targeting control for 48 h before binding (**B**) a H3N2 (A/Burlington/UVM-1927/2022) or a (**C**) A/Burlington/UVM-0478/2022 as above. To measure cell surface sialic acid levels, A549 cells were first treated with siRNAs as above, after which trypsinized single-cell suspensions were stained with the following biotinylated lectins: (**D**)SNA (targeting alpha-2,6 sialic acids), (**E**) MALI (alpha-2,3 sialic acids), or (**F**)MALII (alpha-2,3 sialic acids). Biotinylated lectins were detected with FITC-streptavidin, and a 7-AAD viability stain was used to identify the live population. Flow cytometry was used to analyze cell surface levels and determine the geometric mean for each lectin. Mean +/–SEMis plotted, normalized to the average of the NT control in each biological experiment (*[*P*<0.05]).*N*=4from two biological experiments (**A**),*N*=8from three biological experiments (**B**),*N*=11–12from two biological experiments (**C**),and *N*=5from two biological experiments (**G and H**).Statistical comparisons were done using the Welch’s *t*-test (**A**)and the Brown-Forsythe and Welch ANOVA tests (**B, C, G, I, H**).

To explore our hypothesis that Rab11B was acting at the earliest stages of entry, we used an RT-qPCR-based assay to examine how the rate of binding was affected by depletion of Rab11B. We adapted an assay that we have previously used to measure levels of Hantavirus binding (46), in which we synchronized viral binding to the surface of cells by binding at 4°C to prevent endocytosis. We then washed cells to remove unbound virions and used RT-qPCR to measure the viral genome copies of UVM-1927 (H3N2) attached to cells after 1 h. To verify that we were measuring authentic bound virus (rather than virus sticking non-specifically to cells or plastic), we used exogenous neuraminidase (NA) to cleave bound virus, as NA plays a key role in cleaving sialic acids to release newly produced virions that would otherwise remain attached to the producer cell (21). Using this control, we found a significant drop in viral binding, with ~97% of viral RNA removed upon NA treatment (Fig. 8A). Having confirmed we were detecting authentically bound virions, we next conducted the binding experiment with UVM-1927 in cells lacking Rab11A or Rab11B. Notably, in cells lacking Rab11B, we observed a significant (~50%) decrease in binding compared to cells treated with a NT control, while binding of virions in cells depleted of Rab11A was unaffected (Fig. 8B). Next, we repeated this experiment using the UVM-0478 (H1N1) virus to confirm that the binding defect was specific to the scenario in which viral protein production failed to occur. In this scenario, cells lacking Rab11B had no defect in viral binding (Fig. 8C), suggesting that the ability of Rab11B to support viral binding is specific to H3N2 viruses and is dispensable for the binding of this H1N1 isolate.

Finally, we wanted to determine if Rab11B was altering the global levels of sialic acid present on the cell surface, given the importance of sialic acid in influenza virus binding. To investigate this, we used biotinylated lectins targeting α2,6 (SNA) or α2,3 (MALI and MALII) sialic acids displayed on the cell surface in cells treated with siRNAs targeting Rab11A, Rab11B, or a nontargeting control (Fig. 8D through F). We observed no significant change in the geometric mean of cell surface sialic acid expression in cells lacking Rab11B for any of the lectins measured (Fig. 8G and H).

## DISCUSSION

**Fig 9:**
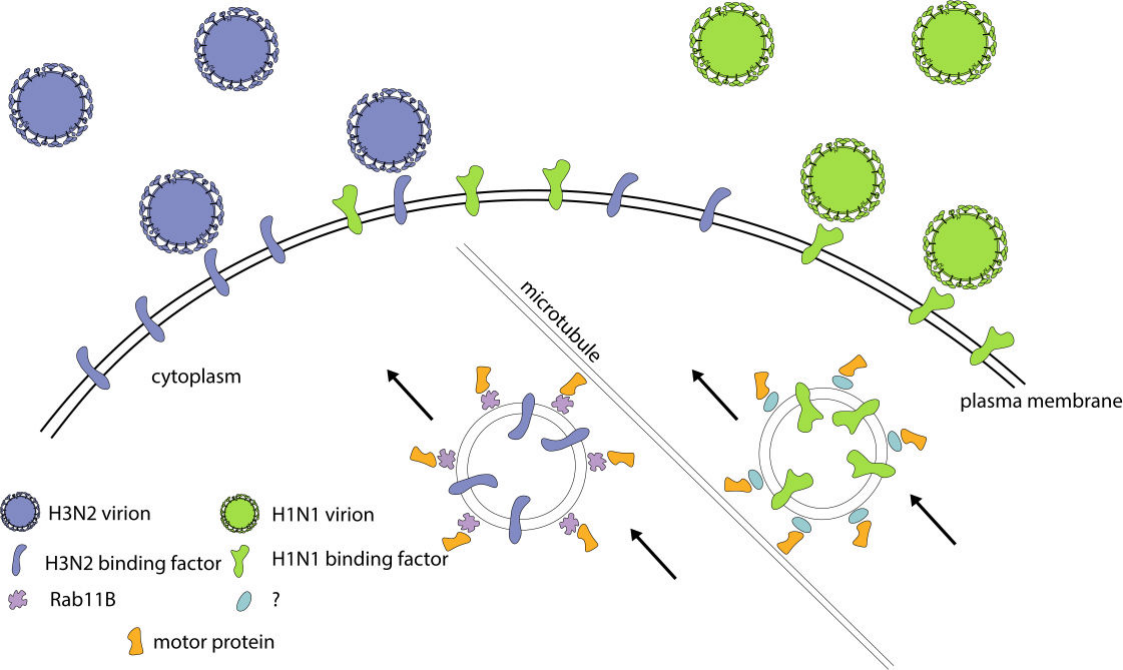
Rab11B may transport a H3N2-specific IAV binding receptor. We propose a model in which H3N2 virions (blue) bind to different cell surface proteins than H1N1 virions (green), and these cell surface proteins (putative binding “receptors”) are differentially transported by the Rab11B recycling pathway. In this framework, protein(s) required for H3N2 virions to bind are transported on Rab11B-positive vesicles, while the molecule(s) required for H1N1 binding are transported by a different (as of yet unidentified) cellular pathway that is independent of Rab11B.

The data presented here are consistent with a model in which recently circulating H1N1 and H3N2 influenza subtypes bind to different cellular receptors or attachment factors. In this framework, the H3N2 receptor(s) is trafficked by Rab11B, while the H1N1 receptor(s) is transported by a non-Rab11A/B dependent mechanism (Fig. 9). While we were initially surprised to observe such different roles for these two Rab11 isoforms in the context of influenza infection, prior literature does support the isoforms playing distinct and even opposing functions within the cell, despite their sequence similarity (42, 43). To some degree, the distinct functions of Rab11A and Rab11B are a result of differential expression, where Rab11A is ubiquitously expressed while Rab11B is enriched in the brain, testis, and heart (while having a lower but broad level of expression in other tissues) (43). In many cases, Rab11A and Rab11B can transport the same cargo, functioning in a redundant manner (47).

However, there are also examples of Rab11A and Rab11B playing distinct roles within the same cellular background. Rab11B plays a role in trafficking fibroblast growth factor receptor 4 (FGFR4) that is different and distinct from Rab11A’s role in this pathway (48), and Rab11B but not A has been implicated in transport of the cystic fibrosis transmembrane conductance regulator (CFTR) (49). Notably, Rab11B (but not Rab11A) is critical for recycling the protease-activated receptor-1 (PAR1), while enhanced degradation of PAR1 observed in cells lacking Rab11B is blocked by simultaneous depletion of Rab11A (43). This phenotype is similar to what we observed in our initial infection of cells with UVM-1927 (our H3N2 subtype), where depletion of Rab11B alone blocked viral protein production but simultaneous depletion of Rab11A + Rab11B rescued the phenotype (Fig. 2E; Fig. S3). While Rab11A and Rab11B share a high degree of sequence similarity, the crystal structures of the two isoforms reveal potentially significant differences, as Rab11A is seen as a dimer in the crystal structure, while Rab11B crystallized as a monomer (50). In addition, there were significant differences in the confirmation of the Switch I region, which is of particular interest given the likely role Rab11A’s Switch I region plays in binding to PB2 (30, 50).

While it seems clear that Rab11A and Rab11B can transport distinct pools of proteins to the plasma membrane, the fact that Rab11B traffics a protein (or proteins) that is required for binding of H3N2 but not H1N1 subtypes of influenza virions is particularly interesting, given our current understanding of influenza entry. Conventional thinking of influenza entry typically posits that influenza virions “roll” along the cellular surface, interacting with sialoglycans present on various glycoproteins and glycolipids that mediate the first step, “attachment.” After this binding event, more recent work proposes that glycan-binding is followed by secondary engagement of the receptor, followed by internalization (18). Within this framework, it has long been recognized that the linkage type and subsequent “shape” of sialic acid modifications play important roles in the ability of a given HA to bind to a particular host cell, with α2,6-linked structures forming an “umbrella”-like structure preferred by human IAVs, while α2,3 linkages form a “cone”-like shape that is preferentially recognized by strains of avian origin (51–58).

Importantly, further modifications to the glycan also affect glycan topology, and it is known that not every glycan with an α2,6 linkage is recognized by every influenza strain that possesses α2,6 receptor specificity (18). While some studies report relatively similar glycan usage between seasonal H1N1 and H3N2 isolates, H3N2 isolates often bind a smaller subset of glycans (58, 59). H3N2 isolates are known to be particularly sensitive to passage in eggs, with H3N2 isolates developing increased specificity for α2,6 linkages, which has posed a problem for egg-grown vaccine development (60). In addition, there are non-sialic acid attachment factors that can affect HA binding, such as phosphorylated but non-sialylated glycans (61). The H17N10 and H18N11 influenza subtypes (found in South and Central American bats) cannot bind sialic acid (62–64) and instead use the MHC-II receptor (65). Furthermore, H2N2 viruses from both human and avian origin have dual receptor specificity and are able to utilize either a sialicacid-dependent or a sialic acid-independent, but MHC-II-dependent, mechanism of entry (66).

Some studies have also shown that specific cell surface proteins have the ability to interact with HA in a sialic acid-independent manner, including nucleolin (67) and natural killer (NK) cell p46-related protein (NKP46) (68–70). Other work concludes that a specific sialylated protein is responsible for HA binding and subsequent internalization, with the voltage-dependent CA^2+^ channel Ca_v_1.2 reported to play this role for PR8 (8). Engagement of cellular signaling receptors, including phosphatidylinositol-3-kinase and receptor tyrosine kinases (RTKs), including the epidermal growth factor receptor (EGFR), has also been shown to play a role in transmitting signals across the plasma membrane that lead to internalization (9–14). Finally, there are reports of subtype-specific dependence on cellular entry factors, with post-binding internalization of H1N1 but not H3N2 subtypes depending on the phospholipase C-γ1 (PLC-γ1) signaling mediator downstream of receptor tyrosine kinase pathways (15). As much prior work has focused on binding and entry of H1N1 strains (typically using the highly lab-adapted PR8 strain), it is likely that further subtype-specificities in cellular binding and internalization receptors remain to be identified.

Our data clearly establish the subtype-specific role of Rab11B in the binding of recent seasonal H3N2 but not H1N1 isolates. However, as Rab11B is not a transmembrane protein, it almost certainly plays an indirect role in this process by trafficking a protein or proteins required for H3 binding to the cell surface. Given the abundance of proteins in the surface proteome with an α2,6 linkage, it seems somewhat unlikely that Rab11B is solely responsible for trafficking the entire variety of proteins conventionally thought to serve as the binding receptor for human IAV strains. This is supported by our data showing that α2,6 and α2,3 sialic acid surface expression in A549s is unchanged by Rab11B depletion (Fig. 8G through I).Further arguing against a scenario in which the loss of Rab11B simply reduces cell surface levels of sialylated proteins is the fact that entry and binding of H1N1 isolates from the same time period are unaffected by the Rab11B depletion. Our data, therefore, support the existence of a more specific protein-based bindingreceptor that is differentially used by H3N2 isolates from recent years (Fig. 9). Open questions that remain the subject of future study include determining whether the Rab11B-dependent cargo that is transported to the surface is responsible for mediating internalization of the virion in addition to binding. Mapping the plasma membrane resident cargos of the Rab11B-dependent recycling pathway could provide a valuable starting point in identifying putative attachment and/or internalization receptors used specifically by recent H3N2 IAVs. Future work is also needed to determine whether these findings extend to older H3N2 isolates, as well as whether this observation is true in cell types of other origins and species. In summary, this work extends the well-established dependence of IAV on Rab11A late in infection to include recent seasonal H1N1 and H3N2 isolates while also extending our understanding of the critical role this protein family plays to include Rab11B-mediated binding and entry of H3N2 viruses.

## MATERIALS AND METHODS

### Cells

Human adenocarcinoma 549 cells (A549) (kindly obtained from Dr. Steve Baker), human embryonic kidney cells (HEK-293T/17) (kindly provided by Dr. John Salogiannis), human papillary adenocarcinoma lung cells, considered “club-like cell” (NCI-H441, ATCC HTB-174), and Madeline Darby kidney cells modified to express α2,6 sialic acid and TMPRSS2 (71) (MDCK-SIATT) (kindly provided by Dr. Jesse Bloom via Dr. Steve Baker) were cultured in 1× DMEM (Corning #06923002) with 10% FBS (Gibco #16140-071) and 1× pen/strep (Corning #30-002-CI). NCI-H441 cells stably transduced with Rab11B targeting shRNA were maintained in media as above, plus 3 μg/mL puromycin.

### Viruses

A/Burlington/UVM-1927/2022 (H3N2) (UVM-1927) and A/Burlington/UVM-0478/2022 (H1N1) (UVM-0478) viruses were isolated from IAV-positive clinical specimens graciously provided by Dr. Jessica Crothers at the University of Vermont. MDCK-SIATT cells were seeded in a 12-well plate (CytoOne #CC7682-7512) at a density of 3.5×10^5^ cells per well 24 h before isolation. On the day of isolation, each clinical sample was brought up to a total volume of 400µL in serum-free media (Corning #10-017-CM). Plated cells were washed twice in PBS (Corning #21-040-CV), and 400µl of the sample was placed in each well. Cells were incubated for 1h at 37°C and 5% CO_2_, rocking the plates every 20 min during the incubation. After 1h, the supernatants were removed from each well and replaced with 1mL media comprised of Opti-MEM (Gibco #11-058-021) + 1µg/mL TPCK-Trypsin (Sigma-Aldrich #T88002-100MG) + 1× pen/strep (Corning #30-002-CI). Samples were deemed ready to be collected when visible cytopathic effect (CPE) could be seen. At that time, supernatants were clarified at 10,000×*g* for 5 min, transferred to clean tubes, and stored at −80°C for later stock generation. Low-passage stock of A/Baltimore/JH-0586/2022 (H3N2) (JH-0586) virus (EPI_ISL_16766241) originally isolated in human nasal epithelial cells was a kind gift of Dr. Andrew Pekosz at Johns Hopkins University. A/Puerto Rico/8/1934 (H1N1) was derived by reverse genetics.

### Generation of 7+1 reassortants by reversegenetics

Viruses containing the HA or NA segments of A/Burlington/UVM-1927/2022 (H3N2) in a A/Puerto Rico/8/1934 (H1N1) backbone were created by reverse genetics, using a pHW2000 plasmid system kindly provided by Dr. Stacey Schultz-Cherry. The HA or NA coding sequence (based on sequencing of the UVM-1927 stock) was synthesized and cloned into the pHW2000 HA or NA segment plasmid (replacing the original PR8 coding sequence) by Genscript. PR8 is an attenuated lab strain that does not cause human disease and has a long history of safe use (72, 73). HEK-293T cells were seeded in six-well plates (7×10^5^ cells/well) in complete DMEM. The following day, cells were transfected with 450ng of each of the eight pHW2000 plasmids (PB1, PB2, PA, HA, NP, NA, M, and NS) using jetOPTIMUS (for per reaction volumes of 3.5 µg combined segment DNA, 50 µL jetOPTIMUS buffer, 3.5 µL jetOPTIMUS, incubated for 10 min before dropwise addition to adherent HEK-293T cells); 24 h post-transfection,the media were removed, and HEK 293T cells were overlaid with 3×10^5^ MDCK-SIATT cells/well resuspended in 2 mL OPTI-VGM (optiMEM, 0.19% BSA, 1× Pen/Strep, 1 µg/mL TPCK Trypsin). Cells were monitored every 24 h for cytopathic effect compared to a transfection control lacking PB2. Once CPE was observed, the rescued virus was blind passaged by infecting a confluent T25 of MDCK-SIATT cells with 500 µL clarified rescue supernatant combined with 500 µL OPTI-VGM, 2 µg/mL TPCK-trypsin for 3 days. Blind passages were titered by plaque assay and used to generate additional high-titer stocks.

### Viral stocks

Viral stocks were grown by seeding 4.2×10^6^cells/flask of MDCK-SIATTs in a T150 flask (Corning #430825) in 1× DMEM (Corning #06923002) with 10% FBS (Gibco #16140-071) and 1× pen/strep (Corning #30-002-CI) and incubated overnight at 37°C and 5% CO_2_. Each flask was washed twice with PBS (Corning #21-040-CV) to remove serum and infected at an MOI of 0.001 in a volume of 5 mL for 1 h, overlaid with 15 mL of Opti-MEM (Gibco #11058-021) plus 1 µg/mL TPCK trypsin (Sigma-Aldrich #T88002-100MG) and incubated at 37°C until ~50%CPE was observed.

### Viral infections

Viral infections were performed at the indicated MOI, by first washing the cells once with PBS (Corning #21-040-CV) and then infecting with an inoculum made up of virus plus 1× DMEM (Corning #06923002) at 37°C and 5% CO_2_ for 1 h. Cells were rocked every 20 min, and after 1 h, the inoculum was removed and replaced with an overlay of Opti-MEM (Gibco #11058-021) before incubating for the indicated time at 37°C. Viral supernatants were clarified by centrifuging at 10,000×*g* for 5 min to remove any cell debris, aliquoted into two separate tubes, and stored at −80°C.

### Viral plaqueassays

Infectious viral production was assessed using plaque assays. MDCK-SIATT cells were trypsinized using 0.25% trypsin-EDTA (Gibco #25200-0720) for 10–15 min and seeded at 1×10^6^cells/well of a six-well plate (Corning #3506). The following day,the wells were washed with PBS (Corning #21-040-CV) or DMEM (Corning #06923002) and inoculated with serial 10-fold dilutions of each sample in DMEM. After infection, the cells were placed at 37°C with 5% CO_2_ for 1 h and rocked every 15 min to ensure even mixing and prevent cell death from drying. After 1 h, the cells were overlayed with 2 mL of a warmed 1:1 mixture of 2.4% Avicel RC-591 NF (Dupont #RC591-NFDR080) + 1× DMEM (Corning #06923002) + 1µg/mL TPCK trypsin (Sigma-Aldrich #T88002-100MG). Cells were returned to 37°C for 48 h, and care was taken not to disturb the plates during this period. Finally, cells were washed with PBS (Corning #21-040-CV), fixed with 4% formaldehyde (Honeywell #F16354L) for 20 min and stained with 0.1% crystal violet (Fisher #C581-100) for 5 min. Plates were rinsed three times with water and allowed to dry before plaques were counted to determine viral titer.

### Viral sequencing

Viral subtypes were identified through amplicon-based whole genome sequencing. In brief, RNA from viral stocks was extracted using a QIAamp Viral RNA Mini Kit (Qiagen #52906) according to the manufacturer’s protocols, and one-step RT-PCR was used to amplify all eight segments using universal primers (74) (Table 1) and Superscript III high-fidelity RT-PCR kit (Invitrogen #12574-035).

**TABLE 1:** Sequencing primers used for RT-PCR in this study

Name/target	Company	Sequence
Uni12/Inf-1_IAV (74)	IDT	5′-GGGGGGAGCAAAAGCAGG-3′
Uni12/Inf-3_IAV (74)	IDT	5′-GGGGGGAGC**G**AAAGCAGG-3′
Uni13/Inf-1_IAV (74)	IDT	5′-CGGGTTATTAGTAGAAACAAGG-3′

Per reaction, 8.75 µL nuclease-free water, 12.5 µL of 2× RT-PCR buffer, 0.2 µL of 10 µM Uni12/Inf-1, 0.3 µL of 10 µM Uni12/Inf-3, 0.5 µL of 10 µM Uni13/Inf-1, and 0.25 µL RT/Taq enzyme mix were combined with 2.5 µL of RNA template. RT-PCR was run on an Eppendorf Mastercycler Nexus Thermal Cycler with the following cycling conditions: 55°C/2 min; 45°C/60 min; 94°C/2 min; 5 cycles of 94°C/30s, 44°C/30s,and 68°C/3.5 min; 26 cycles of 94°C/30s, 57°C/30s, 68°C/3.5 min; 68°C/10 min, and hold at 4°C.

Successful amplification was confirmed by visualizing PCR products (i.e.,7–8 bands) on a 1% agarose (Fisher #BP160-500) gel for 2 h at 120V. Samples were then purified using a QIAquick PCR Purification Kit (Qiagen #28104), and Illumina-based amplicon sequencing was conducted by the Emory EPC Genomics Core.

We used a Nextflow pipeline developed by Dr. Ramiro Barrantes Reynolds at the Vermont Integrative Genomics Resource Core. This pipeline uses Nextflow/NF-Core-based UPHL-BioNGS/Walkercreek v2.0.0 bioinformatics pipeline for analysis and adds a custom Nextflow pipeline for generating a phylogenetic tree (75–81). We compared the similarity between our two H3N2s by using an RShiny app developed to examine the results of the pipeline described above and understand the sequence-level differences between the two viruses. We identified their relatedness through the tree created by the pipeline described above (82). All R coding was done in RStudio (83) (R2023.09.1+494), and we used Nextflow 24.04.4 for pipeline development. Computation was done using the Vermont Advanced Computing Center (VACC).

### siRNA knockdowns

A549 cells were seeded in 24-well plates at a density of 1.5×10^5^cells/well, in a volume of 500 µL. Transfections were performed on cells in suspension or within 24 h of plating. To knockdown our gene of interest, we transiently transfected siRNA sequence targeting Rab11A, Rab11B, Rab11A+B, Rab8A, or a non-targeting siRNA, using Lipofectamine RNAiMAX Reagent (Invitrogen #56532) in A549 cells (Table 2). For each well, we combined 1.2 µL of 10 µM siRNA stock with 198.6 µL of Opti-MEM (Gibco #11058-021) and 2 µL of RNAiMax (Invitrogen #56532), gently mixed, and allowed to rest for 20 min before adding to cells dropwise; 48 h post-transfection, the cells were either infected or RNA was harvested to verify mRNA knockdown at the time of infection.

**TABLE 2:** siRNA reagents used in this study

siRNA target	Company, catalog no.	Assay ID
Non-targeting control	ThermoFisher, 4390843	NA
Rab11A	ThermoFisher, 4390824	s16704
Rab11B	ThermoFisher, 4390824	s17648
Rab8A	ThermoFisher, 4390824	s8681

### shRNA depletion

#### Lentivirus generation

A custom lentiviral construct driving inducible shRNA expression targeting human Rab11B was obtained from Horizon Discovery (SMARTvector-inducible human Rab11B mCMV-Turbo RFP shRNA). Lentiviral particles were generated by transfecting subconfluent HEK-293T cells with 6 µg of the Rab11B SMARTvector construct and 28.5 µg of the Horizon Trans-Lentiviral shRNA packaging kit (Horizon Discovery, cat #TLP5912) using jetOPTIMUS (Sartorius Item #101000025) according to the manufacturer’s instructions. Cells were incubated for 16 h before transfection media were changed to reduced serum media (1× DMEM containing 5% FBS, 1× Pen/Strep). For each of the following 3 days, the supernatant was collected and replaced with fresh media. The supernatants were clarified and stored at −80°C for use in subsequent transductions.

#### Lentiviral transduction

H441 cells were seeded at 1.5×10^5^cells/well in a 24-well plate and transduced the following day with lentiviral supernatants by spinnoculation. H441 cells were inoculated with 500 µL total volume (consisting of complete DMEM, 0.5 μL polybrene, and 100μL of lentiviral supernatant). Cells were spun for 2 h at 640×*g*, 20°C, after which cells were returned to the incubator. The following day, the media were replaced, and the cells were returned to the incubator. Three days post-transduction, cells were selected with puromycin (3 μg/mL), the dilution in which ~30%of cells survived was maintained in puromycin-containing media and expanded for future use.

#### Silencing and validation

Rab11B depletion was optimized by comparing a range of doxycycline concentrations (0.1 μg, 0.5 μg, 2 μg, and 4 μg) and times post-induction (24, 48, and 72 h). Rab11B silencing was measured by RT-qPCR; cells were lysed in 350 μL of RLT buffer for RNA extraction and RT-qPCR as described below. The optimal time and concentration were found to be 4 μg/mL of doxycycline 72 h post-induction;hence, this was used for all further shRNA depletion experiments.

### RNA extraction

After 48 hof transfection, cells treated with siRNAs were lysed in 350 µL of RLT lysis buffer (from RNeasy Mini Kit). Cell lysates were processed, and RNA was extracted using the QIAshredder columns (Qiagen #79656), followed by RNeasy Mini Kit (Qiagen #74106) coupled with on-column DNase treatment using RNase-Free DNase Set (Qiagen #79256), according to the manufacturer’s protocols.

### RT-qPCR

We used a 20 µL reaction consisting of 5 µL of New England Biolabs Luna Probe One-Step RT-qPCR 4× Mix with UDG (NEB #M3019E), 1 µL of the 20× primer/probes stock, 1 µL of extracted RNA, and 13 µL of nuclease-free water for all of our RT-qPCR with pre-mixed primer/probes (Table 3). To measure levels of IAV, we used the SVIP-MPv2 primers and probe (84). We again used 20 µL reaction volume with 5 µL of New England Biolabs Luna Probe One-Step RT-qPCR 4× Mix with UDG (NEB #M3019E), 0.28 µL each of 100 µM forward and reverse primer stock, 0.08 µL of 100 µM probe stock, 13.36 µL of nuclease-free water, and 1 µL of template RNA. Each sample was run in triplicate, and each primer/probe set had a no-template control. Once loaded into plates (Applied Biosystems #4306737), samples were spun at 400 ×*g* for 5 min. RT-qPCR was run using a QuantStudio Real-Time PCR system using the following cycling conditions: 55°C/10 min; 95°C/1 min; 45 cycles of 95°C/10s and 60°C/30s, and 4°C hold. Relative RNA levels were determined using the ΔΔC_T_ method, using 18S RNA levels as the housekeeping control.

**TABLE 3:** RT-qPCR primers used in this study

Target	Company, catalog no.	Assay ID/sequence
18S	ThermoFisher, 4331182	Hs99999901_s1
Rab11A	ThermoFisher, 4331182	Hs00366449_g1
Rab11B	ThermoFisher, 4331182	Hs00188448_m1
Rab8A	ThermoFisher, 4331182	Hs00180479_m1
SVIP-MPv2 (84)	IDT	F: 5′-GGCCCCCTCAAAGCCGA-3′ R: 5′-CGTCTACGYTGCAGTCC-3′ P2-MGB: 5′-TCACTKGGCACGGTGAGCGT-3′ (Label: 5′-FAM-3′MGB-NFQ(EQ))

### Protein analysis

Samples were prepared for protein analysis and western blot largely as previously published(85). Briefly, cells were lysed in a NP-40 (Alfa Asear #J60766) + 1% Triton X-100 (Fisher #BP151-100) supplemented with Pierce Protease Inhibitor Mini Tablets (Thermo Scientific #A32955) for 20 min on ice. Cell lysates were clarified, then diluted 1:1 (vol/vol) with 4× Laemmli sample buffer (250 mM Tris-HCl pH 6.8, 40% glycerol, 8% SDS, 0.04% Bromophenol Blue, 2.75mM 2-mercaptoethanol). All samples were heated to 95°C for 5–10 min before loading. Samples were separated in NuPAGE 4%–12% Bis-Tris gels (Invitrogen # NPO335BOX) in MES buffer (Invitrogen #NP0002) with a molecular mass ladder (Thermo Fisher #LC5925) at 180 V for 50 min before being transferred into a nitrocellulose membrane (Invitrogen #IB23001) using an iBlot 2 machine (Invitrogen) at 20V for 7 min.

Membranes were blocked in a 5% milk/PBS solution for 30 min and then incubated overnight at 4°C in a solution containing the primary antibody (Table 4) in a 5% milk/PBST (PBS + 0.2% Tween 20). Membranes were washed thricefor5 min in PBST, incubated while rocking with secondary antibodies diluted in 5% milk/PBST for 45 min, washed again with PBST, and imaged with a LI-COR Odyssey CLx. Protein expression was analyzed by measuring band densitometry in the LI-COR software package, Image Studio (Ver 5.5).

**TABLE 4:** Primary and secondary antibodies used in this study

Species, target	Company, catalog no.	Dilution used
Primary antibodies		
Rabbit anti-HA	Sino Biological, 86001-RM01	1:5,000 (western blot) 1:1,000 (flow cytometry)
Mouse anti-M2 (14C2)	Abcam,AB5416-1001	1:5,000 (western blot) 1:1,000 (flow cytometry)
Mouse anti-NP (HB65)	BioXCell,BE0159R001MG	1:1,000 (western blot)
Anti-GAPDH	Abcam,AB181602	1:10,000 (western blot)
Rabbit anti-Udorn (H3N2)	From Dr. Robert Lamb	1:1,000 (flow cytometry)
Secondary antibodies		
IRDye 680LT goat anti-rabbit IgG	LICOR,92668021	1:10,000
IRDye 680RD goat anti-mouse IgG	LICOR,92668070	1:10,000
IRDye 800CW goat anti-mouse IgG	LICOR,92632210	1:10,000
Alexa Fluor 488 anti-rabbit	Invitrogen,A11034	1:1,000
Alexa Fluor 647 anti-mouse	Invitrogen,2555690	1:1,000

### Measuring infection by flowcytometry

Infected cells were washed in PBS, trypsinized, and fixed in a final concentration of 4% paraformaldehyde (Thermo Scientific #043368.9M) for 20 min. After fixing, cells were transferred to 5 mL polystyrene round-bottom tubes (Falcon #352052) and spun at 400× *g* for 5 min to pellet cells. Supernatants were discarded, and cells were resuspended in 4 mL of wash buffer (1× PBS/5% FBS). Cells were pelleted, and 200 µL of PBS/0.05% Triton X-100 (Fisher #BP151-100) solution was added to permeabilize for 5 min. Cells were pelleted and washed as above before resuspending in 1 mL of wash buffer for 1 h to block. Cells were pelleted and resuspended in 200 µL of primary antibody solution (diluted in wash buffer) for 1 h (see Table 4). Cells were pelleted and washed as above before resuspension in 200 µL of secondary antibody solution (again diluted in wash buffer) for 1 h (protected from light). Cells were washed and pelleted one final time, after which they were resuspended in 200 µL of sterile PBS and stored at 4°C protected from light until flow cytometry was performed. Samples were run on a Beckman CytoFlex (2L/4 fluorescences) in the Harry Hood Bassett Flow Cytometry and Small Particles Detection facility (RRID:SCR_022147) at the UVM-LCOM and analyzed using FloJo software (v10.10.0).

### Binding assay

Cells that had been transfected with siRNA 48 h earlier were placed on ice to cool, washed with ice-cold PBS, and infected with virus at an MOI of 1 (diluted in a binding buffer of Opti-MEM, 10 mM HEPES,10 mM MES to prevent changes in pH during incubation without CO_2_). Cells remained on ice (to prevent endocytosis), the virus was bound for 1 h and rocked every 15 min to ensure cells received an equal distribution of viral inoculum and prevent cells from drying. Cells were then washed three times with ice-cold PBS to remove unbound virions. At this point, the cells were either lysed in 350 µL RLT buffer (for RNA extraction and analysis of bound virions) or used for the time of addition assay. Alternatively, cells were treated with 0.0043 U/µL of exogenous neuraminidase (Roche #11585886001) for 20 min at 37°C to strip-bound virions from the cell surface (21). Cells were washed three times with PBS after neuraminidase treatment before lysis in 350 µL RLT as above.

### Time of addition

Using a protocol adapted from one recently described by the Chlanda lab (44), we first synchronized infection using the binding protocol described above. After the initial hour of viral binding, cells were overlaid with Opti-MEM and were placed at 37°C. At 0, 20, 45, 60, or 90 min, the Opti-MEM was removed and replaced with Opti-MEM containing 15 mM ammonium chloride (NH_4_Cl). Cells were then incubated at 37°C for 16 h, before preparing for flow cytometry as described above.

### Alpha 2,3/2,6-sialyation profiling by flow cytometry

Protocol was adapted from Z Biotech “Alpha 2,3/2,6 Sialyation Profiling Kit User Manual” (Z Biotech #10902-20T). A549 cells were seeded and knocked down as described above, with all per-well values doubled as 12-well plates were used instead. At 48 h post-transfection, the cells were washed once with 1× PBS and then placed in 500 µL 0.25% Trypsin-EDTA per well and incubated at 37°C with 5% CO2 until cells detached from wells. Cells were then transferred to 5 mL tubes (same as above) with 1 mL of 1× DMEM (Corning #06923002) with 10% FBS (Gibco #16140-071) and 1×Pen/Strep (Corning #30-002-CI). Cells were then spun at 400 ×*g* for 5 min, and the supernatants were discarded. Cells were then placed in 1 mL of block (1× PBS with 5% FBS), gently vortexed, and incubated at 4°C for 1h. Then, ~3mL of 1× PBS was added to each tube, and cells were spun at 400 ×*g* for 5 min. Supernatants were discarded, and lectins were added at 1 µg/100 µL in cell staining buffer (Z Biotech #10902-20T). Cells were incubated on ice for 1 h, vortexing every 15 min. Then, ~4mL of wash buffer (1×PBS + 1%FBS) was added to each tube;the cells were spun at 400×*g* for 5 min, and supernatants were discarded. Cells were then vortexed, and then the wash was repeated. Then, cells were placed in 0.25 µg/100 µL of FITC-Streptavidin (Invitrogen #11431787) and incubated at 4°C for 1 h, vortexing every 15 min. Cells were then washed as after initial lectin staining, and samples were transferred to a 96-well plate, and 5 µL of 7-AAD (Invitrogen #00699350) was added as a live-dead stain. Samples were run on MACSQuant and analyzed using FloJo software (v10.10.0).

## Data Availability

Raw sequencing data are available in the NCBI Bioproject database under ID PRJNA1254704.

## SUPPLEMENTAL MATERIAL

Supplemental material jvi.02111-25-s0001.pdf Figures S1 to S3.

ASM does not own the copyrights to Supplemental Material that may be linked to, or accessed through, an article. The authors have granted ASM a non-exclusive, world-wide license to publish the Supplemental Material files. Please contact the corresponding author directly for reuse.
