# Supplementary material for: Rab11Bis required for binding and entry of recent H3N2, but not H1N1, influenza A isolates
Source: J Virol. 2026 Jun 2;100(6):e02111-25. doi: 10.1128/jvi.02111-25 (PMC13289146; doi:10.1128/jvi.02111-25)
Supplement: Supplemental material — Figures S1 to S3. [file jvi.02111-25-s0001.pdf]

Fig. S1

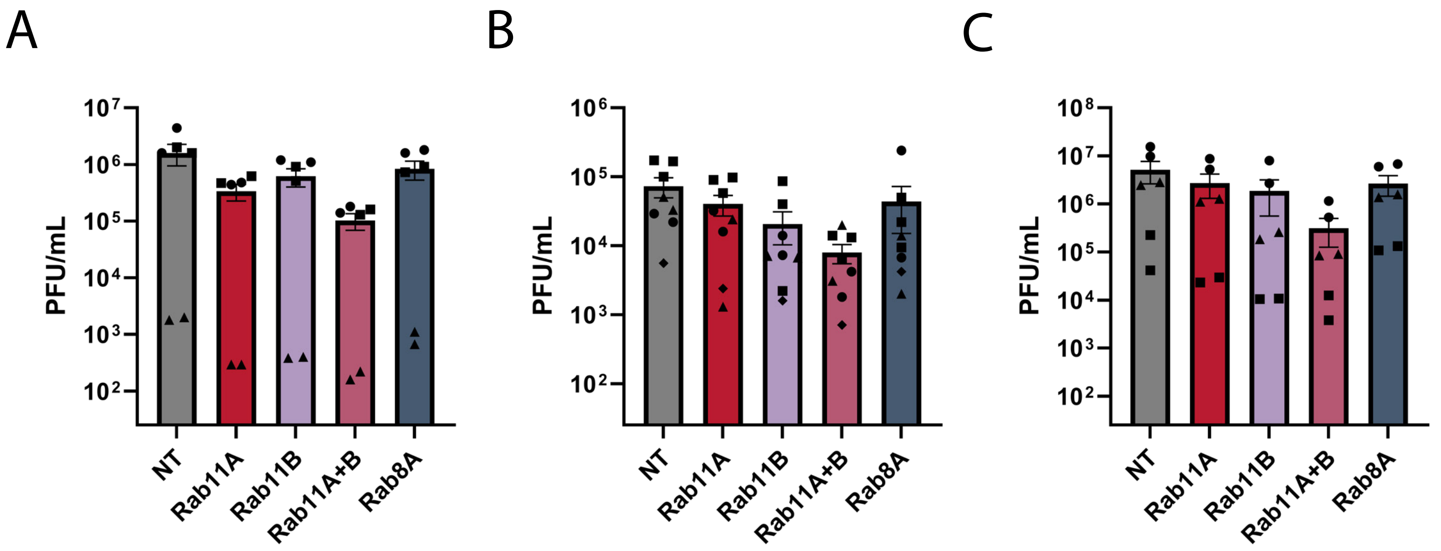

Figure S1. Raw titers of viral production in cells infected with recent H1N1 and H3N2 influenza A isolates show Rab11A and Rab11B are consistently required for infectious virus production in both subtypes.

Raw titer data from cells infected with **A)** UVM-0478 (H1N1) [Figure 1B], **B)** UVM-1927 (H3N2) [Figure 1C], or **C)** JH-0586 (H3N2) [Figure 2A] is presented here as PFU/mL. Biological replicates are distinguished by shape (■, ◆, ▲ , or ●) with N=6 from three biological experiments (A, C) and N=8 from four biological experiments (B).

Fig. S2

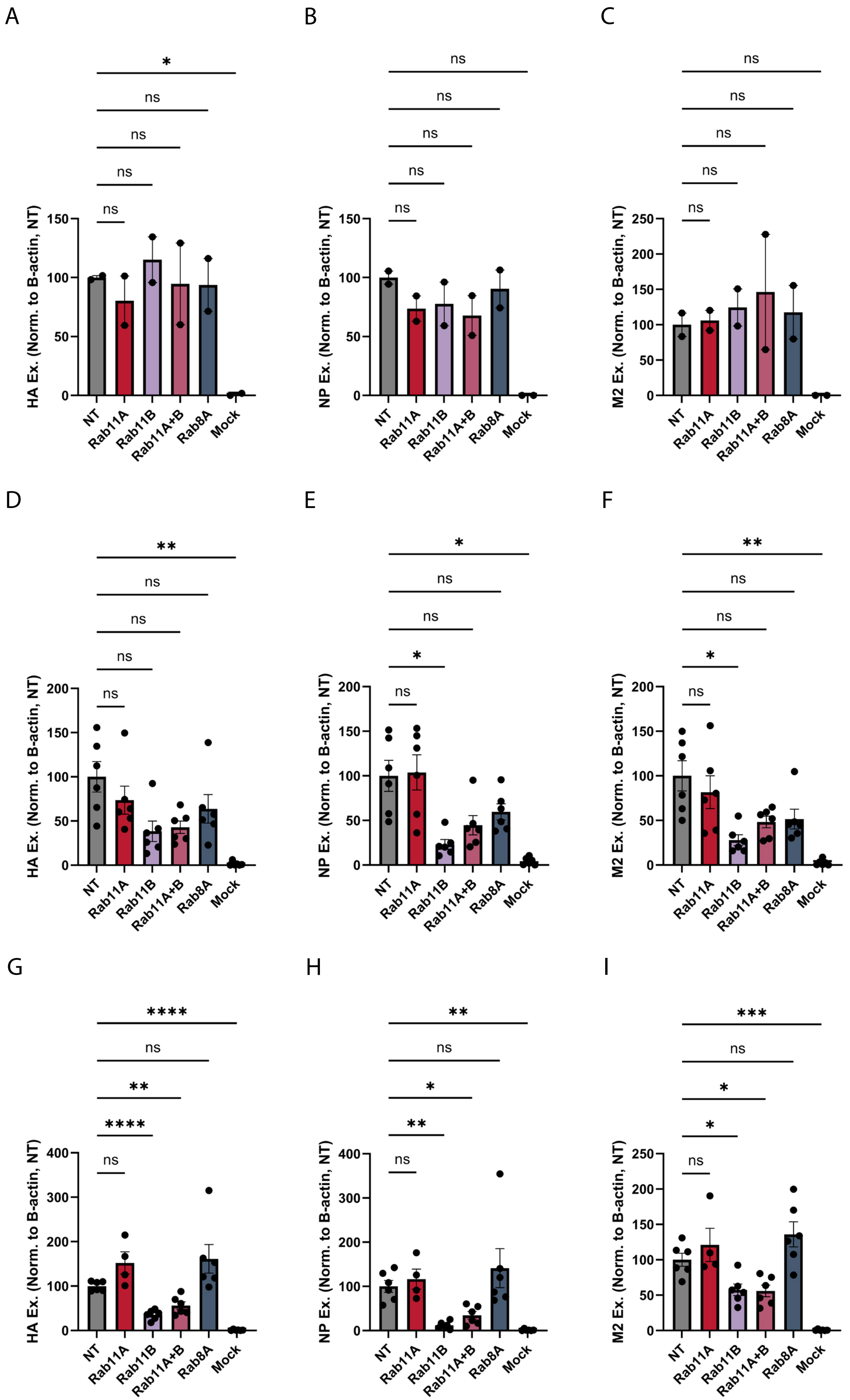

A
B
C
D
E
F
G
H
I
HA Ex. (Norm. to B-actin, NT)
NP Ex. (Norm. to B-actin, NT)
M2 Ex. (Norm. to B-actin, NT)
NT
Rab11A
Rab11B
Rab11A+B
Rab8A
Mock
ns
*
**
***
****

Figure S2. The dependence of H3N2 viral protein production in cells lacking Rab11B is seen with normalization to multiple cellular housekeeping genes.

Data presented in Figure 2 and Figure 3 was alternatively quantified here by normalizing to β-actin and the average of NT control for each biological replicate; **(A)** Figure 2B, **(B)** Figure 2C, **(C)** Figure 2D, **(D)** Figure 2F, **(E)** Figure 2G, **(F)** Figure 2H, **(G)** Figure 3C, **(H)** Figure 3D, and **(I)** Figure 3E is shown for HA0 **(A, D, G)**, NP **(B, E, H)** and M2 **(C, F, I)**. Mean +/- SEM is plotted, N=2 from one biological experiment **(A-C)** and N=6 from three biological experiments **(D-I).** Statistical comparisons done using Welch's one way ANOVA with Dunnett's multiple comparisons [*($p<0.05$), **($p<0.01$), ***($p<0.001$), ****($p<0.0001$)].

Fig. S3

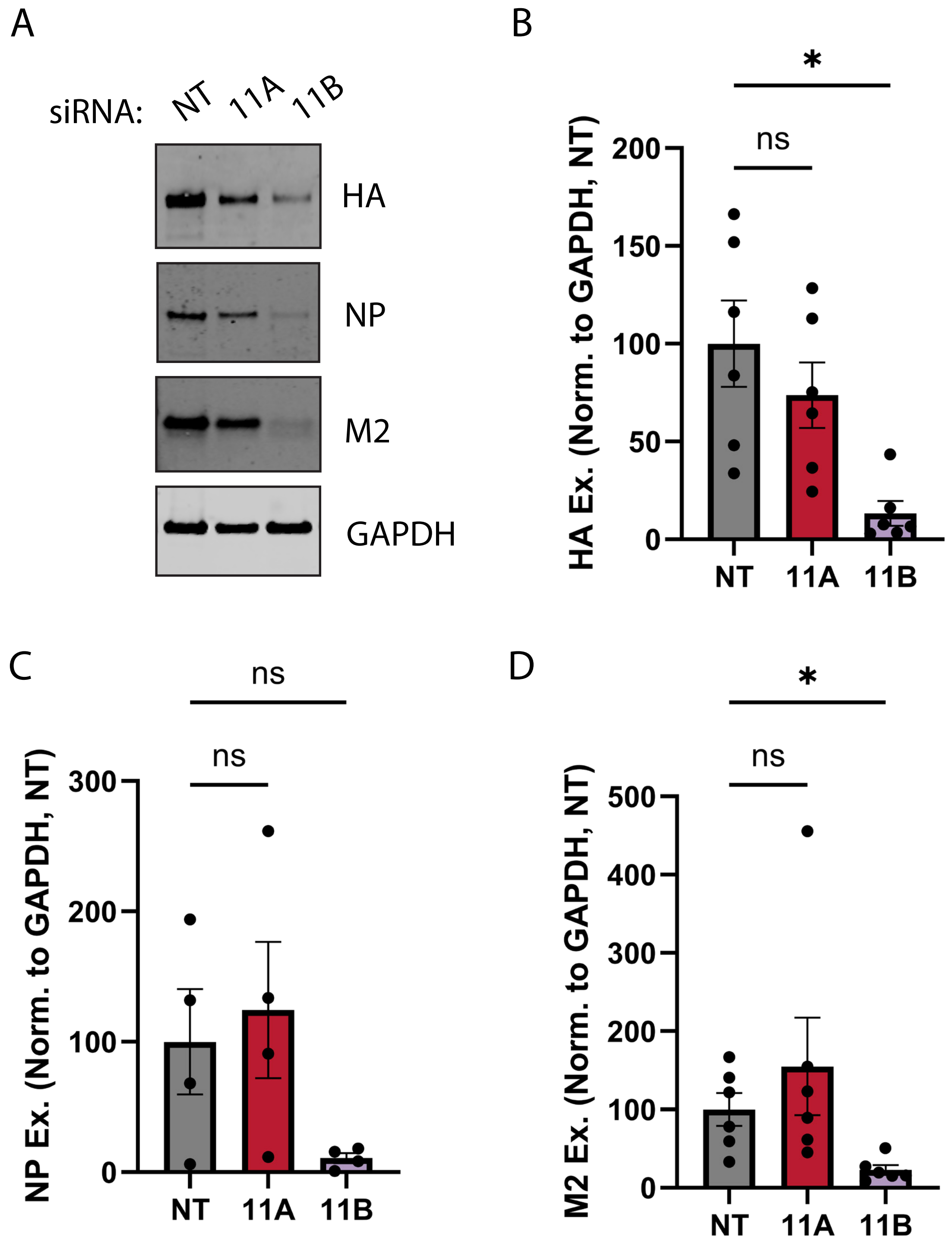
A
siRNA:
NT
11A
11B
HA
NP
M2
GAPDH
B
*
ns
HA Ex. (Norm. to GAPDH, NT)
200
150
100
50
0
NT
11A
11B
C
ns
ns
NP Ex. (Norm. to GAPDH, NT)
300
200
100
0
NT
11A
11B
D
*
ns
M2 Ex. (Norm. to GAPDH, NT)
500
400
300
200
100
0
NT
11A
11B

Figure S3. Rescue of Rab11B-dependent defect in H3N2 viral protein production in cells simultaneously depleted of Rab11A and Rab11B is not an artifact of knockdown efficiency.

A549 cells were treated with siRNAs targeting Rab11A, Rab11B or a non-targeting control at half the amount typically used (5nm instead of the standard 10 nm). 48 hpt cells were infected with UVM-1927 at an MOI of 1, or mock infected. **A)** 16 hpi cell lysates were collected and proteins visualized by SDS-PAGE and western blot using rabbit anti-HA and anti-GAPDH antibodies in addition to mouse anti-NP and anti-M2 antibodies. Expression of viral proteins was quantified and normalized to GAPDH levels and the average of the NT control for each biological replicate is shown for HA0 **(B)**, NP **(C)** and M2 **(D)**. Mean +/- SEM is plotted, N=6 from three biological experiments. Statistical comparisons done using Welch's one way ANOVA with Dunnett's multiple comparisons (*=$p<0.05$).
